# Ancient role of vasopressin/oxytocin-type neuropeptides as regulators of feeding revealed in an echinoderm

**DOI:** 10.1186/s12915-019-0680-2

**Published:** 2019-07-31

**Authors:** Esther A. Odekunle, Dean C. Semmens, Nataly Martynyuk, Ana B. Tinoco, Abdullah K. Garewal, Radhika R. Patel, Liisa M. Blowes, Meet Zandawala, Jérôme Delroisse, Susan E. Slade, James H. Scrivens, Michaela Egertová, Maurice R. Elphick

**Affiliations:** 1 School of Biological & Chemical Sciences, Queen Mary University of London, Mile End Road, London, E1 4NS UK; 2 Waters/Warwick Centre for BioMedical Mass Spectrometry and Proteomics, School of Life Sciences, University of Warwick, Coventry, CV4 7AL UK; 3 Department of Clinical Neurosciences, MRC Cambridge Stem Cell Institute, University of Cambridge, Cambridge, CB2 0SZ UK; 4 Department of Neuroscience, Brown University, Providence, USA; 5 Research Institute for Biosciences, Biology of Marine Organisms and Biomimetics, University of Mons (UMONS), 7000 Mons, Belgium; 6 Waters Corporation, Stamford Avenue, Altrincham Road, Wilmslow, SK9 4AX UK; 7 School of Science, Engineering & Design, Teesside University, Stephenson Street, Tees Valley, TS1 3BA UK

**Keywords:** Echinoderm, Vasopressin, Oxytocin, Asterotocin, Asterotocin receptor, mRNA in situ hybridisation, Immunohistochemistry, Cardiac stomach, Feeding, Righting

## Abstract

**Background:**

Vasopressin/oxytocin (VP/OT)-type neuropeptides are well known for their roles as regulators of diuresis, reproductive physiology and social behaviour. However, our knowledge of their functions is largely based on findings from studies on vertebrates and selected protostomian invertebrates. Little is known about the roles of VP/OT-type neuropeptides in deuterostomian invertebrates, which are more closely related to vertebrates than protostomes.

**Results:**

Here, we have identified and functionally characterised a VP/OT-type signalling system comprising the neuropeptide asterotocin and its cognate G-protein coupled receptor in the starfish (sea star) *Asterias rubens*, a deuterostomian invertebrate belonging to the phylum Echinodermata. Analysis of the distribution of asterotocin and the asterotocin receptor in *A. rubens* using mRNA in situ hybridisation and immunohistochemistry revealed expression in the central nervous system (radial nerve cords and circumoral nerve ring), the digestive system (including the cardiac stomach) and the body wall and associated appendages. Informed by the anatomy of asterotocin signalling, in vitro pharmacological experiments revealed that asterotocin acts as a muscle relaxant in starfish, contrasting with the myotropic actions of VP/OT-type neuropeptides in vertebrates. Furthermore, in vivo injection of asterotocin had a striking effect on starfish behaviour—triggering fictive feeding where eversion of the cardiac stomach and changes in body posture resemble the unusual extra-oral feeding behaviour of starfish.

**Conclusions:**

We provide a comprehensive characterisation of VP/OT-type signalling in an echinoderm, including a detailed anatomical analysis of the expression of both the VP/OT-type neuropeptide asterotocin and its cognate receptor. Our discovery that asterotocin triggers fictive feeding in starfish provides important new evidence of an evolutionarily ancient role of VP/OT-type neuropeptides as regulators of feeding in animals.

**Electronic supplementary material:**

The online version of this article (10.1186/s12915-019-0680-2) contains supplementary material, which is available to authorized users.

## Background

Physiological processes and behaviour are controlled and regulated by a huge variety of neuronally secreted peptide signalling molecules (neuropeptides), which typically exert their effects on target cells by binding to cognate G-protein coupled receptors (GPCRs) [1–3]. Amongst the most intensely studied neuropeptides are vasopressin (VP) and oxytocin (OT). VP and OT are structurally related neuropeptides that were originally discovered based on their peripheral actions as pituitary hormones in mammals. VP is a cyclic and amidated nonapeptide (CYFQNCPRG-NH_2_; with a disulphide bridge between the cysteines) that acts as an antidiuretic and increases blood pressure, whereas OT (CYIQNCPLG-NH_2_) causes uterine contraction and lactation [4]. VP and OT are also released within the brain, and the discovery that they are key players in neural mechanisms of social and reproductive behaviour in humans and other animals has led to a revolutionary growth in interest in these neuropeptides as potential therapeutic agents for the treatment of disorders such as autism, social anxiety and schizophrenia [5].

Phylogenetic analysis has revealed that neuropeptide signalling systems are evolutionarily ancient. Thus, the evolutionary origin of at least 30 neuropeptide families (including VP/OT-type) can be traced back to the bilaterian common ancestor of deuterostomes (vertebrates and invertebrate chordates, hemichordates, echinoderms) and protostomes (e.g. arthropods, nematodes, molluscs, annelids) [3, 6, 7]. Definitive evidence of the antiquity of VP/OT-type neuropeptides was first obtained with the purification and sequencing of VP/OT-type neuropeptides from insect and molluscan species [8, 9]. More recently, comparative analysis of genome/transcriptome sequence data has enabled the discovery of genes encoding VP/OT-type neuropeptides and their putative cognate receptors in many bilaterian animal phyla. Furthermore, this has enabled comparative analysis of the physiological roles of VP/OT-type neuropeptides in species belonging to different phyla [10–12]. Interestingly, this has provided evidence that not only the structures but also the functions of VP/OT-type neuropeptides are evolutionarily conserved. Thus, a VP/OT-type neuropeptide in insects (inotocin) has a VP-like role in regulating urine production [13], whereas a VP/OT-type neuropeptide in the mollusc *Lymnaea stagnalis* has an OT-like role in regulating reproductive physiology [14, 15]. Furthermore, application of reverse genetic techniques in the nematode *Caenorhabditis elegans* revealed that VP/OT-type signalling is required for normal mating behaviour in this species [16], consistent with the actions of VP and OT in regulating mating behaviour and reproductive physiology in mammals [5].

Comparative investigation of the physiological roles of VP/OT-type neuropeptides has largely focused on vertebrates and selected protostomian invertebrates, as highlighted above. What are missing are insights from the Ambulacraria (echinoderms and hemichordates), deuterostomian invertebrates that are more closely related to chordates (including vertebrates) than protostomes [17]. A gene encoding the VP/OT-type neuropeptide echinotocin has been identified in the sea urchin *Strongylocentrotus purpuratus*, and in vitro pharmacological studies revealed that echinotocin acts as a muscle contractant in sea urchins, consistent with the myostimulatory actions of VP and OT in mammals [18]. However, a more comprehensive analysis of the biochemistry, anatomy and physiology of VP/OT-type signalling has yet to be reported for an echinoderm species. To address this issue, here we have performed an extensive experimental analysis of VP/OT-type signalling in an emerging model system for neuropeptide research—the starfish (sea star) *Asterias rubens*. Sequencing of the neural transcriptome of *A. rubens* has enabled the identification of transcripts encoding at least 40 neuropeptide precursor proteins [19]. Furthermore, we have begun to characterise selected neuropeptide systems in *A. rubens* in detail. For example, our recent experimental analysis of gonadotropin-releasing hormone (GnRH)-related neuropeptides in *A. rubens* provided important new insights into the evolution of neuropeptide signalling in the animal kingdom [20, 21]. Using the starfish *A. rubens* as a model system, here we report a detailed functional characterisation of VP/OT-type neuropeptide signalling in an echinoderm.

## Results

### Molecular characterisation of a VP/OT-type signalling system in the starfish *A. rubens*

**Fig. 1:**
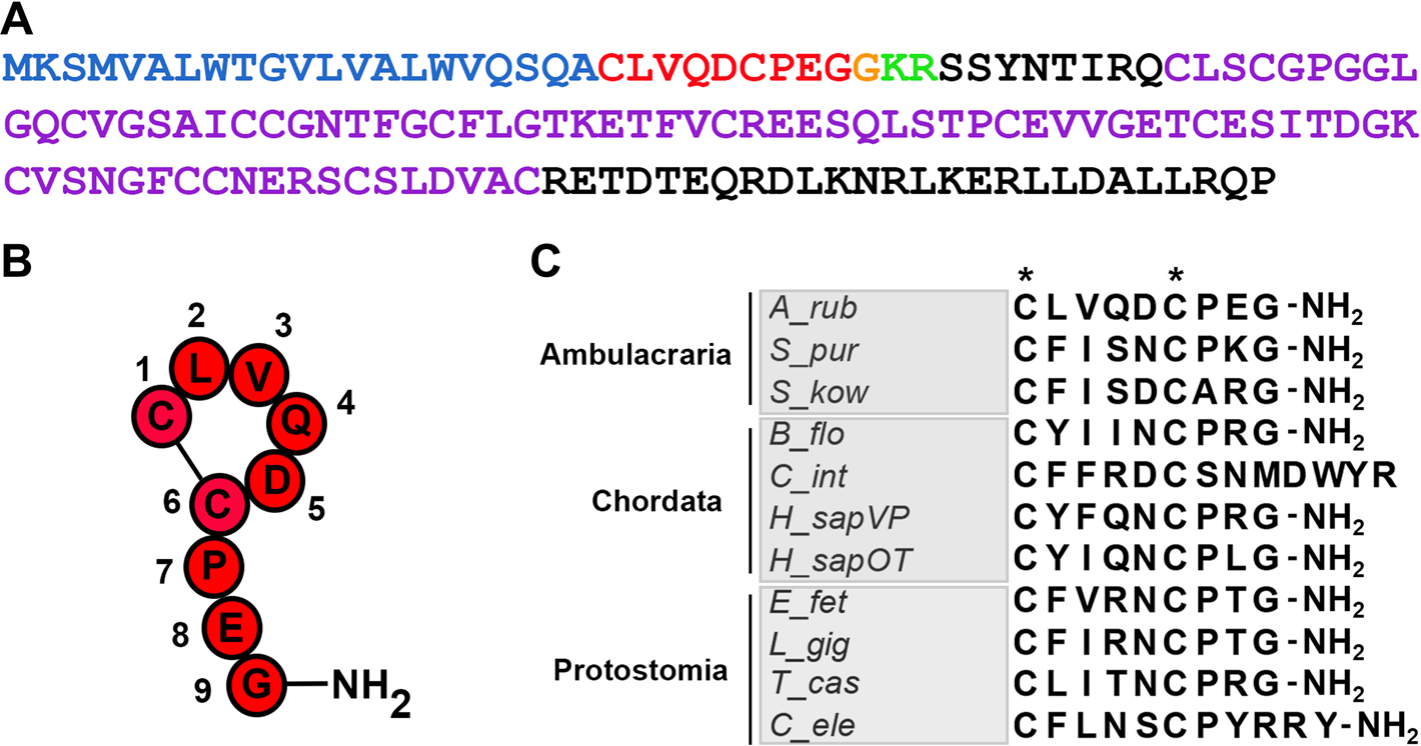
Asterotocin: a vasopressin/oxytocin (VP/OT)-type neuropeptide in the starfish *Asterias rubens*. **a** Amino acid sequence of the *A. rubens* asterotocin precursor with the predicted signal peptide shown in blue, a predicted cleavage site shown in green, the asterotocin peptide shown in red with a C-terminal glycine that is a putative substrate for amidation shown in orange and the neurophysin domain shown in purple. **b** Structure of the mature asterotocin peptide as determined by mass spectrometry (Additional file 1), with a disulphide bridge between the two cysteine residues and with C-terminal amidation. **c** Clustal-X alignment of asterotocin (A_rub) with VP/OT-type peptides from other animals reveals that the pair of cysteine residues (asterisks) is conserved in all of the peptides, whereas other residues are conserved in a subset of the peptides. Full species names within each taxonomic group (Ambulacraria, Chordata and Protostomia) and corresponding accession numbers for the peptide sequences are listed in Table S1 of Additional file 3

A transcript encoding a 147-residue precursor protein comprising an N-terminal signal peptide, a VP/OT-type neuropeptide (asterotocin) and C-terminal neurophysin domain (Fig. 1a), was identified previously by analysis of *A. rubens* neural transcriptome sequence data [19], and its sequence was then confirmed by cDNA cloning and sequencing [22]. Informed by the known structures of VP/OT-type neuropeptides [13, 15, 23, 24], we synthesised the predicted mature asterotocin peptide—CLVQDCPEG-NH_2_, an amidated cyclic nonapeptide with a disulphide bridge between the cysteine residues at positions 1 and 6 (Fig. 1b). Mass spectrometric analysis of extracts of radial nerve cords from *A. rubens* revealed the presence of a peptide with identical properties to synthetic asterotocin (Additional file 1). A comparison of asterotocin with VP/OT-type neuropeptides in other taxa reveals that it is unusual in having an acidic residue (Glu; E) at position 8 (Fig. 1c).

**Fig. 2:**
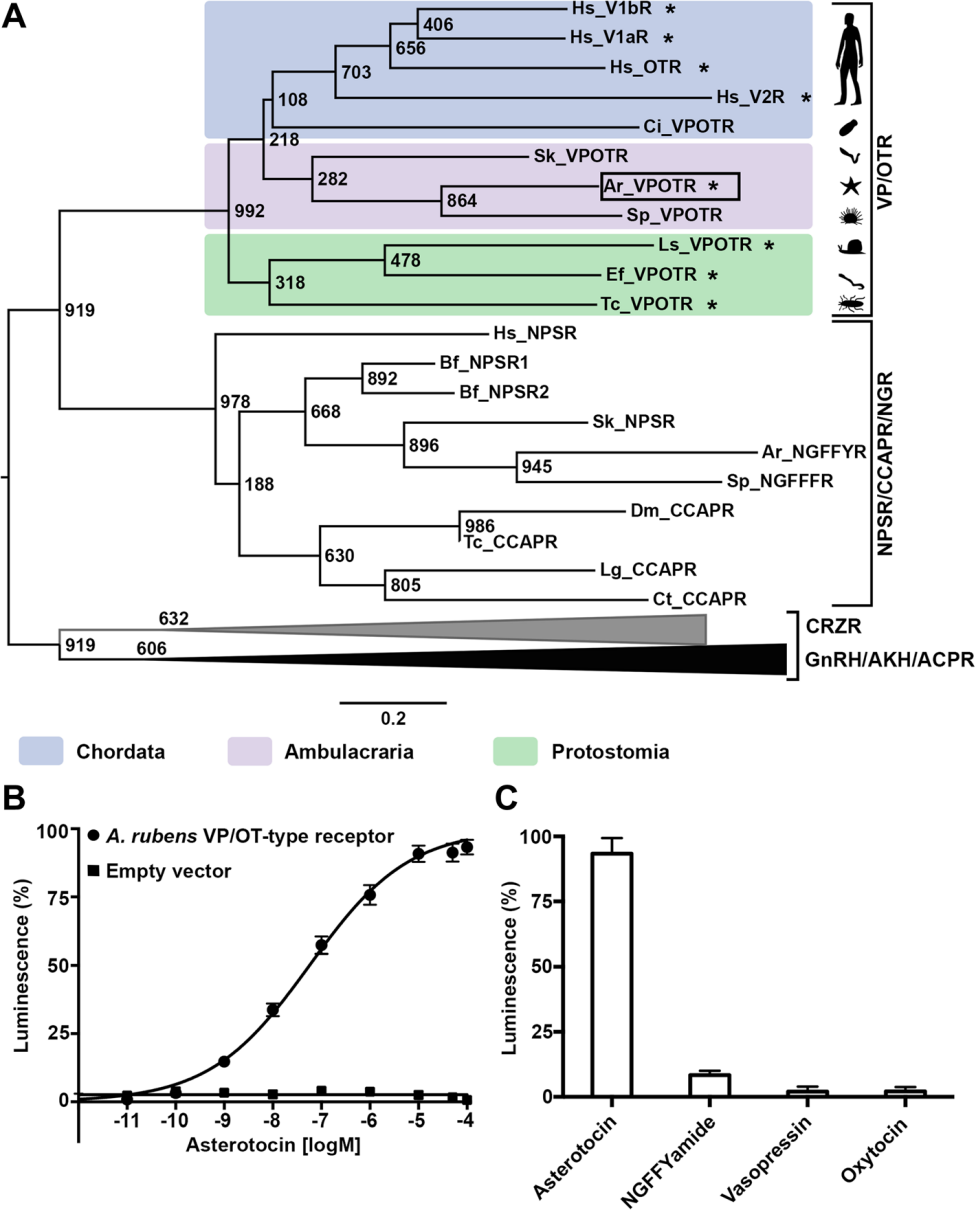
Phylogenetic identification and deorphanisation of an *A. rubens* VP/OT-type receptor. **a** Maximum likelihood tree showing that a VP/OT-type receptor identified by BLAST analysis of *A. rubens* transcriptome sequence data (boxed) is positioned within a clade comprising VP/OT-type receptors from other taxa. NPS/NG peptide/CCAP-type receptors are paralogs of VP/OT-type receptors [25]. Phylogenetic analyses of bilaterian neuropeptide receptors have shown that GnRH/AKH/ACP/CRZ-type receptors are closely related to VP/OT-type receptors and NPS/NG peptide/CCAP-type receptors [6]. Therefore, GnRH/AKH/ACP/CRZ-type receptors were included as an outgroup in the phylogenetic tree. The scale bar indicates amino acid substitutions per site, and bootstrap values are shown at nodes. Species where activation of the VP/OT-type receptor by a cognate VP/OT-type neuropeptide has been demonstrated experimentally (including *A. rubens*, see **b**) are labelled with an asterisk. Full species names and accession numbers for the receptor sequences are listed in Table S2 of Additional file 3. **b** Asterotocin (black circles) causes concentration-dependent activation of the *A. rubens* VP/OT-type receptor, demonstrated by measuring Ca^2+^-induced luminescence in CHO-K1 cells expressing aequorin, and transfected with the promiscuous G-protein Gα16 and the *A. rubens* VP/OT-type receptor (CHO-K1/G5A-ArVPOTR cells); EC_50_ = 5.7 × 10^−8^ M. CHO-K1 cells transfected with empty pcDNA vector (black square) were used as a negative control and do not exhibit luminescence when exposed to asterotocin. **c** Comparison of the relative luminescence responses of CHO-K1/G5A-ArVPOTR cells when exposed to asterotocin or the related peptides NGFFYamide (a paralog of asterotocin in *A. rubens*), human vasopressin or human oxytocin, all at a concentration of 10^−4^ M. Luminescence responses in the presence of vasopressin, oxytocin and NGFFYamide were not significantly higher than the basal media control (*P* = 0.5, *P* = 0.25, *P* = 0.25, respectively; Wilcoxon signed-rank test; *n* = 9)

To identify a candidate receptor for asterotocin, BLAST analysis of *A. rubens* neural transcriptome data was performed using known VP/OT-type receptors from other species as queries. A 2710-bp transcript (contig 1122053) encoding a 428-residue protein was identified, and phylogenetic analysis confirmed that it is an ortholog of VP/OT-type receptors that have been characterised in other taxa (Fig. 2a). A cDNA encoding this receptor was cloned and sequenced (Additional file 2; GenBank accession number MK279533) and then co-expressed with the promiscuous Gα16 protein in Chinese hamster ovary (CHO) cells stably expressing the Ca^2+^-sensitive luminescent apo-aequorin protein. Synthetic asterotocin caused concentration-dependent luminescence in CHO cells transfected with the *A. rubens* VP/OT-type receptor but had no effect on CHO cells transfected with an empty vector (Fig. 2b). The EC_50_ value for asterotocin as a ligand for the receptor was 5.7 × 10^−8^ M, consistent with the potency of VP/OT-type neuropeptides as ligands for their cognate receptors in other taxa [26–29]. We also tested the starfish neuropeptide NGFFYamide, which is a paralog of asterotocin [25, 30, 31], and human vasopressin (VP) and human oxytocin (OT). These three peptides did not cause significant receptor activation, even at the highest peptide concentration tested (10^−4^ M), demonstrating the specificity of the *A. rubens* VP/OT-type receptor for asterotocin as a ligand (Fig. 2c). Having identified molecular components of the VP/OT-type neuropeptide signalling pathway in *A. rubens*, asterotocin and its cognate G-protein coupled receptor, the asterotocin receptor, we then investigated the anatomical distribution of these molecules in *A. rubens* to gain insights into the physiological roles of VP/OT-type signalling in this species.

### The anatomy of starfish

**Fig. 3:**
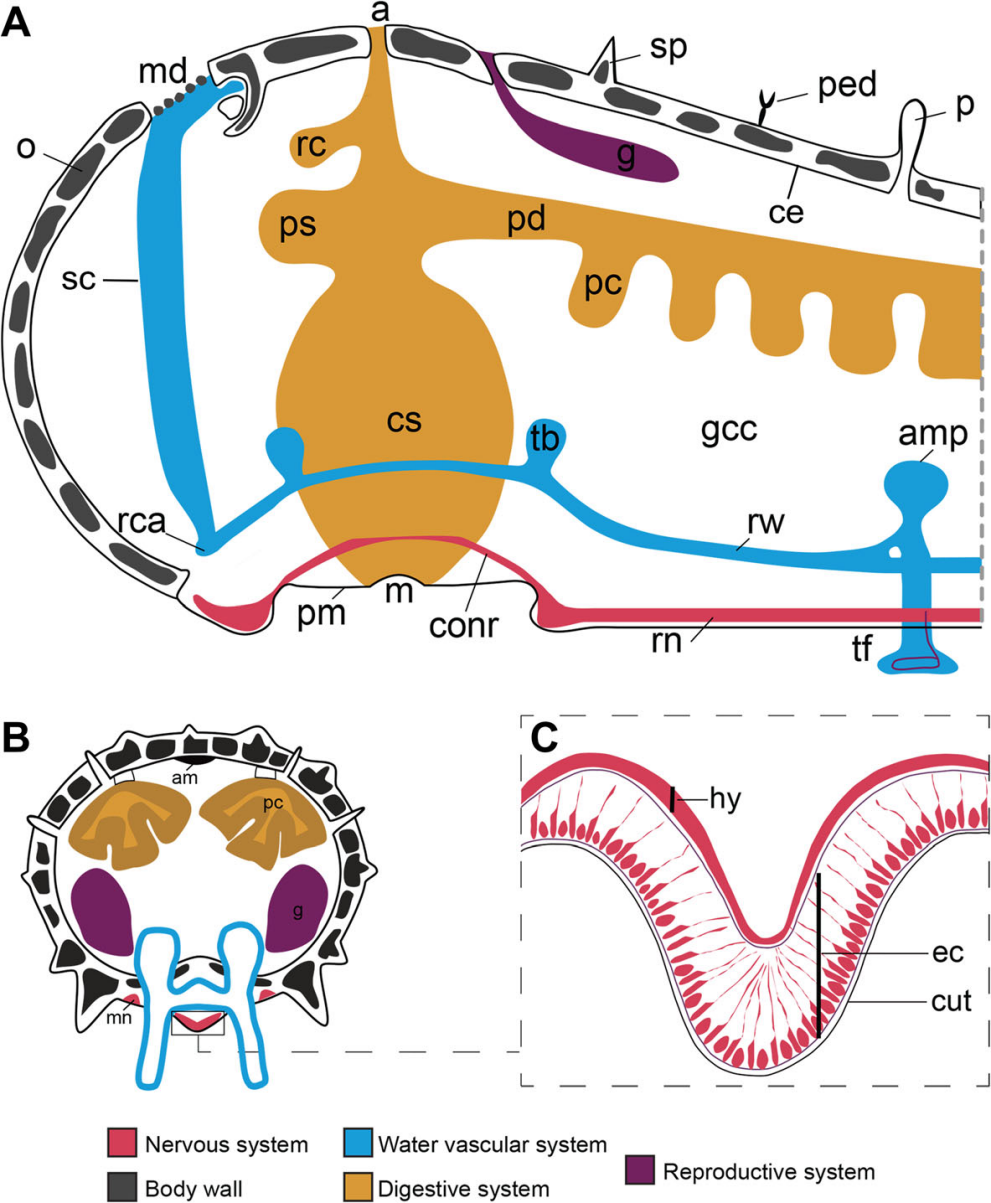
Starfish anatomy. **a** Schematic vertical section of the central disc and the proximal region of an arm. **b** Schematic transverse section of an arm. **c** Schematic transverse section of a radial nerve cord. Abbreviations: a, anus; am, apical muscle; amp, ampulla; conr, circumoral nerve ring; cs, cardiac stomach; cut, cuticle; ec, ectoneural region; g, gonad; gcc, general coelomic cavity; hy, hyponeural region; m, mouth; md, madreporite; mn, marginal nerve; o, ossicle; p, papula; pc, pyloric caecum; pd., pyloric duct; ped, pedicellaria; pm, peristomial membrane; ps, pyloric stomach; rc, rectal caecum; rca, ring canal; rn, radial nerve; rw, radial water vascular canal; sp., spine; sc, stone canal; tb, Tiedemann’s body; tf, tube foot

To facilitate the interpretation of the expression patterns of asterotocin and its receptor in *A. rubens*, in Fig. 3**,** we show schematics of starfish anatomy. The starfish nervous system comprises five radial nerve cords linked by a circumoral nerve ring (Fig. 3a, b), which have two distinct regions (Fig. 3c): the hyponeural region that contains motoneurons and the ectoneural region that is thought to largely comprise sensory neurons and interneurons [32]. Along the length of each arm, flanking the radial nerve cords are rows of tube feet (Fig. 3a, b) that enable locomotor activity. The entrance to the digestive system (mouth) is located on the underside of the central disc region and is surrounded by a contractile peristomial membrane (Fig. 3a). Extending aborally from the mouth is a short tubular oesophagus leading into a large, highly folded and evertible cardiac stomach. Above the cardiac stomach is a smaller pyloric stomach, followed by a short intestine, a rectum (with associated rectal caeca) and a tiny opening (anus) on the aboral surface of the central disc (Fig. 3a). Paired digestive organs located in each arm (pyloric caeca) are connected to the pyloric stomach by pyloric ducts (Fig. 3a, b). Paired reproductive organs (testes or ovaries) are also located in each arm (Fig. 3a, b). The body wall comprises calcite ossicles and associated appendages for defence (pedicellariae and spines) and gas exchange (papulae). The internal surface of the body wall is lined by a coelomic epithelium (Fig. 3a, b), with underlying layers of longitudinally orientated and circularly orientated muscle. In an aboral sagittal position, the longitudinal body wall muscle layer is thickened to form an apical muscle that facilitates arm flexion.

### Localisation of asterotocin precursor transcripts and asterotocin in *A. rubens*

To investigate the distribution of asterotocin precursor transcripts in *A. rubens*, mRNA in situ hybridisation (ISH) methods were employed. To enable immunohistochemical (IHC) visualisation of the mature neuropeptide derived from the asterotocin precursor, we generated a rabbit antiserum to asterotocin (Additional file 4). To determine the specificity of this antiserum, it was tested on the sections of arms from *A. rubens* in parallel with antiserum that had been pre-absorbed with the antigen peptide. The majority of immunostaining observed was abolished by antiserum pre-absorption, but there was some residual staining (Additional file 5). Therefore, antibodies affinity-purified from the antiserum were used for immunohistochemical analysis of asterotocin expression in *A. rubens*.

**Fig. 4:**
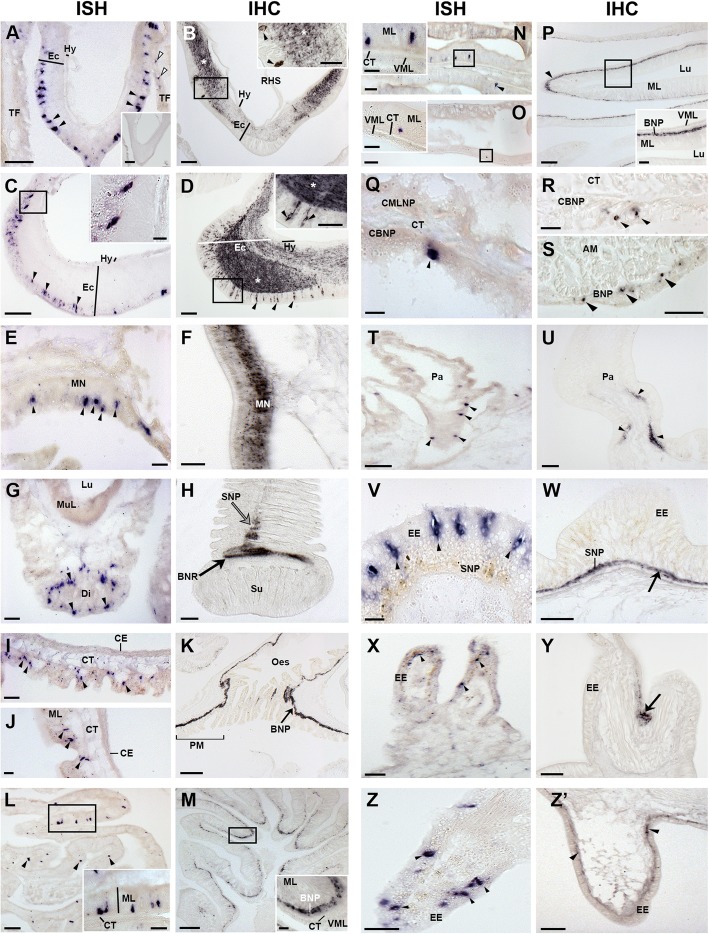
Localisation of asterotocin in *A. rubens* using in situ hybridisation (ISH) and immunohistochemistry (IHC). **a** Asterotocin precursor transcript-expressing cells (AstPtc) in a radial nerve cord (black arrowheads) and tube feet (white arrowheads). Inset shows the absence of staining with sense probes. **b** Asterotocin-immunoreactive (Ast-ir) cells (arrowheads in inset) and fibres (white asterisks) in a radial nerve cord. **c** AstPtc in the circumoral nerve ring (arrowheads and inset). **d** Ast-ir cells (arrowheads) and fibres (white asterisks) in the circumoral nerve ring. **e** AstPtc in marginal nerve. **f** Ast-ir fibres in marginal nerve. **g** AstPtc in tube foot disc. **h** Ast-ir in the basal nerve ring (black arrow) and longitudinal nerve tract (grey arrow) of a tube foot. **i** AstPtc in the peristomial membrane. **j** AstPtc in the oesophagus. **k** Ast-ir in the peristomial membrane (square bracket) and oesophagus. **l** AstPtc in the cardiac stomach. **m** Ast-ir in the cardiac stomach. **n** AstPtc in the pyloric stomach. **o** AstPtc in a pyloric duct. **p** Ast-ir in the pyloric stomach. **q** AstPtc in the coelomic epithelium of the body wall. **r** Ast-ir in the coelomic basiepithelial nerve plexus of body wall. **s** Ast-ir in the basiepithelial nerve plexus of the apical muscle. **t** AstPtc in a papula. **u** Ast-ir in a papula. **v** AstPtc in the body wall. **w** Ast-ir in the body wall. **x** AstPtc in a pedicellaria. **y** Ast-ir in a pedicellaria. **z** AstPtc in a spine. **z’** Ast-ir in an ambulacral spine. Abbreviations: AM, apical muscle; BNR, basal nerve ring; BNP, basiepithelial nerve plexus; CMLNP, circular muscle layer nerve plexus; CBNP, coelomic basiepithelial nerve plexus; CE, coelomic epithelium; CT, collagenous tissue; Di, disc; Ec, ectoneural region; EE, external epithelium; Hy, hyponeural region; LNT, longitudinal nerve tract; Lu, lumen; MN, marginal nerve; ML, mucosal layer; MuL, muscle layer; Oes, oesophagus; Pa, papula; RHS, radial hemal sinus; TF, tube foot; VML, visceral muscle layer. Scale bars: **a**, **a** inset, **h**, **l**, **m**, **n**, **o**, **p**, **u** = 60 μm; **b**, **d**, **g**, **z’** = 40 μm; **i**, **j**, **l** inset, **p** inset, **t**, **w**, **x** = 30 μm; B inset, **c**, **d** inset, **e**, **k**, **n** inset, **o** inset, **s**, **v**, **y**, **z** = 20 μm; **c** inset, **f**, **m** inset, **q**, **r** = 10 μm

In the nervous system, asterotocin precursor-expressing cells and asterotocin-immunoreactive cells were revealed in the epithelial layer of the ectoneural region of the radial nerve cords and circumoral nerve ring, with immunostained processes in the underlying neuropile; no expression was detected in the hyponeural region (Fig. 4a–d). Asterotocin precursor-expressing cells (Fig. 4e) and asterotocin-immunoreactive processes (Fig. 4f) were also revealed in the marginal nerve cords, which are located lateral to the outer row of tube feet in each arm. In the tube feet, asterotocin precursor-expressing cells were revealed in the disc region (Fig. 4g), while asterotocin immunoreactivity was revealed in the basal nerve ring and basal region of the longitudinal nerve tract (Fig. 4h).

Asterotocin precursor-expressing cells were revealed in several regions of the digestive system, including the peristomial membrane (Fig. 4i), oesophagus (Fig. 4j), cardiac stomach (Fig. 4l), pyloric stomach (Fig. 4n) and pyloric duct (Fig. 4o), with stained cells typically located in the upper mucosal layer just below the basiepithelial nerve plexus (see insets of Fig. 4l, n, o). Accordingly, asterotocin immunoreactivity was revealed in the basiepithelial nerve plexus in several regions of the digestive system, including the peristomial membrane and oesophagus (Fig. 4k), cardiac stomach (Fig. 4m) and pyloric stomach (Fig. 4p).

In the body wall, asterotocin precursor-expressing cells and asterotocin-immunoreactive cells were revealed in the coelomic epithelium (Fig. 4q, r), with immunostained processes in the underlying coelomic lining (Fig. 4r). Asterotocin precursor mRNA was not detected in the apical muscle, but immunoreactivity was revealed in the apical muscle (Fig. 4s). Asterotocin precursor-expressing cells were detected in the coelomic lining of papulae (Fig. 4t), and accordingly, asterotocin-immunoreactive processes were revealed in the underlying nerve plexus (Fig. 4u). Additionally, asterotocin precursor-expressing cells are present in the external epithelium of the body wall (Fig. 4v), and associated appendages, including pedicellariae (Fig. 4x) and ambulacral spines (Fig. 4z). Consistent with these findings, asterotocin-immunoreactive fibres were revealed in the basiepithelial nerve plexus of the external body wall (Fig. 4w), pedicellariae (Fig. 4y) and ambulacral spines (Fig. 4z’).

### Localisation of asterotocin receptor expression in *A. rubens*

**Fig. 5:**
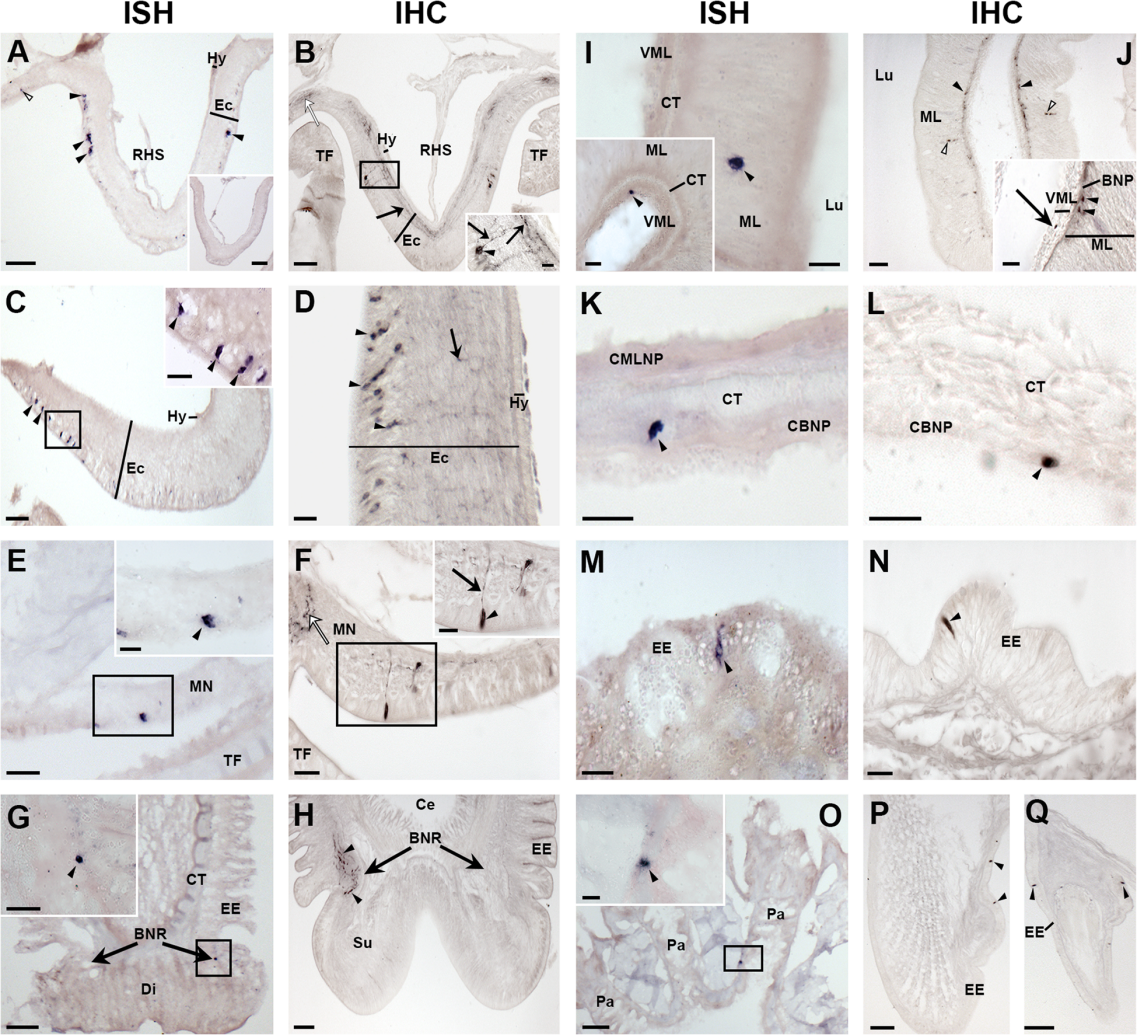
Localisation of asterotocin receptor in *A. rubens* using in situ hybridisation (ISH) and immunohistochemistry (IHC). **a** Asterotocin receptor transcript-expressing cells (AstRtc) in a radial nerve cord (black arrowheads) and tube feet (white arrowheads). Inset shows the absence of staining with sense probes. **b** AstR-immunoreactive (AstR-ir) cells (black arrowhead) and processes (arrows and in inset) in a radial nerve cord. Stained fibres in the adjacent tube foot (white arrow). **c** AstRtc in the circumoral nerve ring. **d** AstR-ir cells (arrowheads) and fibres (arrow) in the circumoral nerve ring. **e** AstRtc in a marginal nerve. **f** AstR-ir cell (arrowhead) and stained process (black arrow) in a marginal nerve. Stained fibres in the adjacent tube foot (white arrow). **g** AstRtc in a tube foot (black arrowhead). **h** Ast-ir in a tube foot. **i** AstRtc in the cardiac stomach. **j** AstR-ir in the mucosal layer (white arrowhead), basiepithelial nerve plexus (black arrowhead) and visceral muscle layer (black arrow; inset) of the cardiac stomach. **k** AstRtc in the coelomic epithelium of the body wall. **l** AstR-ir cell in the coelomic epithelium of the body wall. **m** AstRtc in the external epithelium of the body wall. **n** AstR-ir cell in the external epithelium of the body wall. **o** AstRtc in a papula. **p** AstR-ir cells in an ambulacral spine. **q** AstR-ir cells in a pedicellaria. Abbreviations: BNR, basal nerve ring; BNP, basiepithelial nerve plexus; CMLNP, circular muscle layer nerve plexus; Ce, coelom; CBNP, coelomic basiepithelial nerve plexus; CT, collagenous tissue; Di, disc; Ec, ectoneural region; EE, external epithelium; Hy, hyponeural region; Lu, lumen; MN, marginal nerve; ML, mucosal layer; Pa, papula; RHS, radial hemal sinus; TF, tube foot; VML, visceral muscle layer. Scale bars: **a**, **a** inset, **b**, **c**, **g**, **h**, **o**, **p**, **q** = 60 μm; **d**, **e**, **j** = 30 μm; **c** inset, **f**, **g** inset, **n** = 20 μm; **b** inset, **e** inset, **f** inset, **i**, **i** inset, **j** inset, **k**, **l**, **m**, **o** = 10 μm

Employing the same techniques used to analyse the distribution of asterotocin, localisation of asterotocin receptor expression was similarly determined using in situ hybridisation and immunohistochemistry. For immunohistochemical analysis, a guinea pig antiserum to the asterotocin receptor was generated, and antibodies to the antigen peptide were affinity-purified from the antiserum (Additional file 4). Cell bodies expressing asterotocin receptor mRNA transcripts were revealed in the ectoneural epithelial layer of the radial nerve cords (Fig. 5a) and circumoral nerve ring (Fig. 5c). Consistent with this finding, asterotocin receptor-immunoreactive cells were detected in the ectoneural epithelium, with stained processes in the underlying neuropile of the radial nerve cords (Fig. 5b) and circumoral nerve ring (Fig. 5d). Asterotocin receptor mRNA-expressing cells were revealed in the marginal nerves (Fig. 5e), and accordingly, immunostaining was also observed in cells and processes in the marginal nerves (Fig. 5f). Cells expressing asterotocin receptor mRNA were revealed in tube feet discs (Fig. 5g), and consistent with this finding, immunostained processes were revealed in the basal nerve ring of tube feet, with stained processes also extending into a longitudinal fibre tract that extends aborally from the basal nerve ring (Fig. 5h).

In the digestive system, asterotocin receptor mRNA-expressing cells were revealed in the mucosal layer (Fig. 5i) and the coelomic epithelial lining of the visceral muscle layer of the cardiac stomach (Fig. 5i, inset). Detection of asterotocin receptor immunoreactivity in the cardiac stomach seemed to be sensitive to the decalcification methods used for processing the whole central disc region. Therefore, to avoid the decalcification step, cardiac stomach preparations were fixed in situ and then dissected from the central disc prior to embedding in paraffin wax. Using this method, stained processes were detected in the basiepithelial nerve plexus (Fig. 5j) and in the visceral muscle layer of the cardiac stomach (Fig. 5j, inset).

Asterotocin receptor mRNA-expressing cells were detected in the coelomic epithelium of the body wall (Fig. 5k), and accordingly in Fig. 5l, an asterotocin receptor-immunoreactive cell can be seen in the coelomic epithelium in close proximity to the apical muscle but no asterotocin receptor expression was detected in the apical muscle. Asterotocin receptor mRNA-expressing cells (Fig. 5m) and asterotocin receptor-immunoreactive cells (Fig. 5n) were detected in the external epithelial layer of the body wall. Asterotocin receptor expression was also detected in body wall-associated appendages, including the papulae (Fig. 5o), spines (Fig. 5p) and pedicellariae (Fig. 5q).

**Fig. 6:**
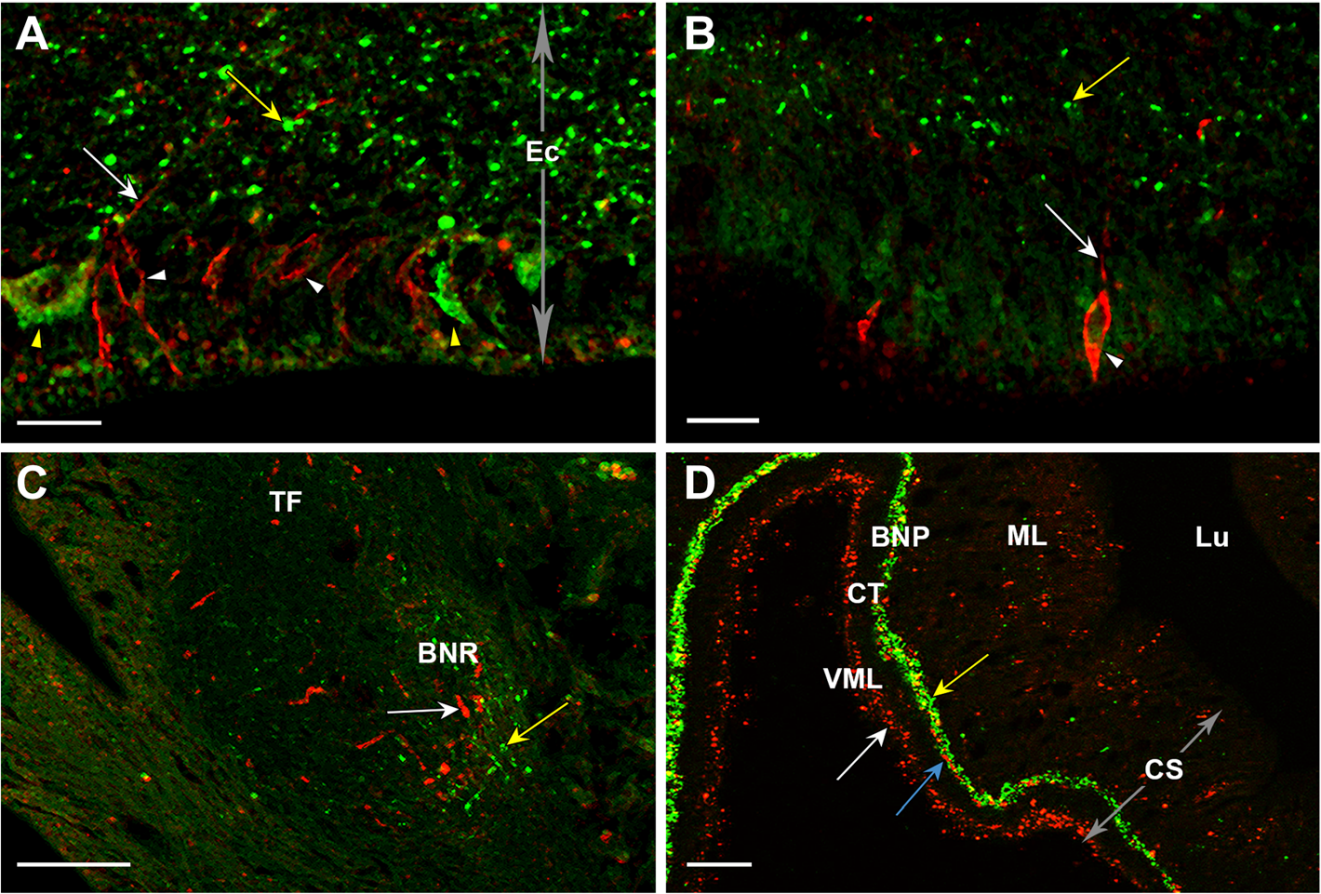
Comparison of asterotocin and asterotocin receptor expression in *A. rubens* using double-labelling fluorescence immunohistochemistry. Comparison of the distribution of asterotocin immunoreactivity (Ast-ir; green) and asterotocin receptor immunoreactivity (AstR-ir; red) in *A. rubens* reveals ‘salt and pepper’ patterns of labelling consistent with expression largely in different, but often adjacent, populations of cells/processes. In the few instances where labelling appears yellow/orange, this could be due to the co-localisation of asterotocin and the asterotocin receptor in processes or alternatively it may simply reflect where asterotocin-containing fibres happen to be positioned directly above asterotocin receptor containing fibres. **a** Radial nerve cord showing Ast-ir cells (yellow arrowheads) and AstR-ir cells (white arrowheads) in the ectoneural epithelium and Ast-ir processes (yellow arrow) and AstR processes (white arrow) in the ectoneural neuropile. **b** Marginal nerve; AstR-ir cells (white arrowhead) in the epithelial layer and both Ast-ir processes (yellow arrow) and AstR-ir processes (white arrow) in the underlying neuropile. **c** Disc region of a tube foot; Ast-ir processes (yellow arrow) and AstR-ir processes (white arrow) in the basal nerve ring. **d** Cardiac stomach; Ast-ir processes (white arrowhead) and AstR-ir processes (blue arrow) in the basiepithelial nerve plexus and mucosa, while in the visceral muscle layer, only AstR-ir is present (white arrow). Abbreviations: BNR, basal nerve ring; BNP, basiepithelial nerve plexus; CS, cardiac stomach; CT, collagenous tissue; Ec, ectoneural; Lu, lumen; ML, mucosal layer; TF, tube foot; VML, visceral muscle layer. Scale bars: **d** = 30 μm; **c** = 20 μm; **a**, **b** = 10 μm

To enable a direct comparison of the distribution of asterotocin immunoreactivity and asterotocin receptor immunoreactivity, double-labelling immunofluorescence methods were employed. This revealed that asterotocin and the asterotocin receptor are expressed in the same regions of the nervous system but appear to be largely expressed in different cells and processes. Thus, in the radial nerve cords, distinct groups of cells exhibiting asterotocin immunoreactivity or asterotocin receptor immunoreactivity can be observed in the ectoneural epithelium. Accordingly, in the ectoneural neuropile, a ‘salt and pepper’ pattern of immunolabelling is observed, with asterotocin-immunoreactive fibres interspersed with less abundant asterotocin receptor-immunoreactive fibres and with little evidence of co-localisation (Fig. 6a). Likewise, in the marginal nerves (Fig. 6b) and basal nerve ring of the tube foot (Fig. 6c), distinct populations of fibres expressing asterotocin or the asterotocin receptor are observed in close proximity. In the cardiac stomach, asterotocin immunoreactivity can be observed in the basiepithelial nerve plexus and mucosa, while asterotocin receptor immunoreactivity can be observed in the basiepithelial nerve plexus, mucosa and visceral muscle layer. As in the radial nerve cords, marginal nerves and basal nerve ring, analysis of immunostaining in the basiepithelial nerve plexus of the cardiac stomach indicates that asterotocin and asterotocin receptor proteins are largely localised in different fibre populations but often in close proximity to each other (Fig. 6d).

With regard to the sub-cellular localisation of the asterotocin receptor, it is noteworthy that asterotocin receptor immunoreactivity can be seen in the cytoplasm of ectoneural cell bodies (Fig. 6b), which probably reflects the presence of receptor proteins in the endoplasmic reticulum and/or Golgi apparatus before they are targeted to the plasma membrane. Functional asterotocin receptors located in the plasma membrane are likely to be present in the processes of cells that express the asterotocin receptor, and accordingly, asterotocin receptor-immunoreactive processes (red) can be seen intermingled amongst asterotocin-immunoreactive processes (green) in the ectoneural neuropile (Fig. 6a, b). However, the resolution of the microscopy was not sufficient to determine if the asterotocin receptor immunoreactivity is specifically associated with the plasma membrane in the tiny cross-sectional profiles of neuronal processes.

### Asterotocin causes relaxation of the cardiac stomach and apical muscle preparations in vitro

**Fig. 7:**
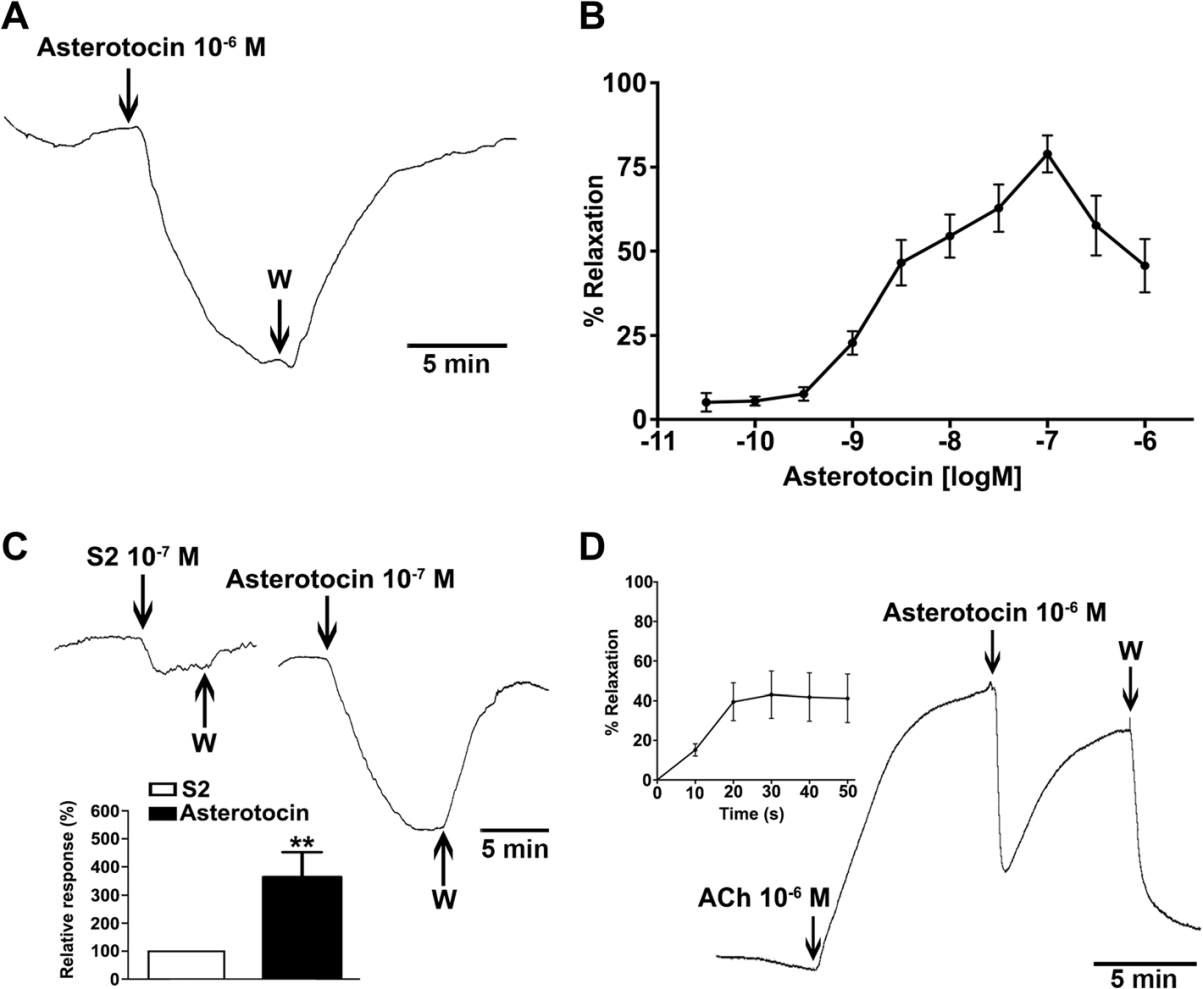
Asterotocin causes relaxation of in vitro cardiac stomach and apical muscle preparations from *A. rubens*. **a** Representative recording showing that asterotocin causes relaxation of a cardiac stomach preparation. Seawater supplemented with KCl (3 × 10^−2^ M) was used to induce contraction of the cardiac stomach prior to the application of asterotocin. The relaxing effect of asterotocin is reversed when the preparation is washed with KCl-supplemented seawater. **b** Graph showing the concentration-dependent relaxing effect of asterotocin on cardiac stomach preparations at concentrations ranging from 3 × 10^−11^ M to 10^−6^ M. The responses are expressed as the mean relative percentage (± SEM; *n* = 16) of the maximal relaxing effect of asterotocin in each preparation. **c** Representative recording from a cardiac stomach preparation that compares the relaxing effects of asterotocin and the SALMFamide-type neuropeptide S2, both at 10^−7^ M. As in **a**, the cardiac stomach preparation was pre-contracted with KCl-supplemented seawater prior to the application of the neuropeptides. Inset compares the effects of asterotocin and S2 on cardiac stomach preparations at a concentration of 10^−7^ M, expressed as mean percentages (± SEM; *n* = 5) with the relaxing effect of S2 defined as 100%. The relaxing effect of asterotocin is significantly larger than the effect S2 (Mann-Whitney *U* test; *P* = 0.0079; *n* = 5). **d** Representative recording showing that asterotocin (10^−6^ M) causes relaxation of an apical muscle preparation. 10^−6^ M acetylcholine (ACh) was used to induce contraction of the apical muscle preparation prior to the application of asterotocin. Following washing of the preparation with artificial seawater, it returns to its basal relaxed state. Inset shows the mean percentage (± SEM; *n* = 4) reversal of 10^−6^ M ACh-induced contraction of apical muscle preparations caused by 10^−6^ M asterotocin over a 50-s period after peptide administration

Both vasopressin and oxytocin cause contraction of the smooth muscle in mammals [4], and we have reported previously that the VP/OT-type neuropeptide echinotocin causes contraction of tube foot and oesophagus preparations from the sea urchin *Echinus esculentus* (Phylum Echinodermata) [18]. Therefore, it was of interest here to investigate the effects of asterotocin on muscle preparations from *A. rubens*. Informed by the expression of asterotocin and its receptor in the cardiac stomach and tube feet, and the localisation of asterotocin immunoreactivity in the apical muscle, asterotocin was tested on in vitro preparations of these three organs. Interestingly, we found that asterotocin causes relaxation of the cardiac stomach and apical muscle preparations (Fig. 7), but it had no observable effect on the tube feet (data not shown). Asterotocin is a potent relaxant of the cardiac stomach, with the peptide causing reversal of KCl-induced contraction at concentrations as low as 3 × 10^−11^ M. The largest mean relaxing effect was observed at ~ 10^−7^ M, with desensitisation often observed at higher concentrations (Fig. 7a, b). Previous studies have revealed that the SALMFamide neuropeptide S2 (SGPYSFNSGLTF-NH_2_) also acts as a relaxant of cardiac stomach preparations in *A. rubens* [33, 34], and therefore, it was of interest to compare the efficacy of asterotocin and S2. Testing both peptides at a concentration of 10^−7^ M, it was observed that the magnitude of the relaxing action of asterotocin was ~ 3–4 times larger than the effect of S2 (Fig. 7c; raw data for the graph are available in Additional file 6: Table S1). In contrast to the potency of asterotocin as a relaxant of cardiac stomach preparations, the relaxing effect of asterotocin on apical muscle preparations was only observed with high peptide concentrations (1 μM; Fig. 7d), with maximal relaxation occurring within 20 s of application (Fig. 7d, inset; raw data for the graph are available in Additional file 6: Table S2).

### Asterotocin triggers cardiac stomach eversion in vivo

**Fig. 8:**
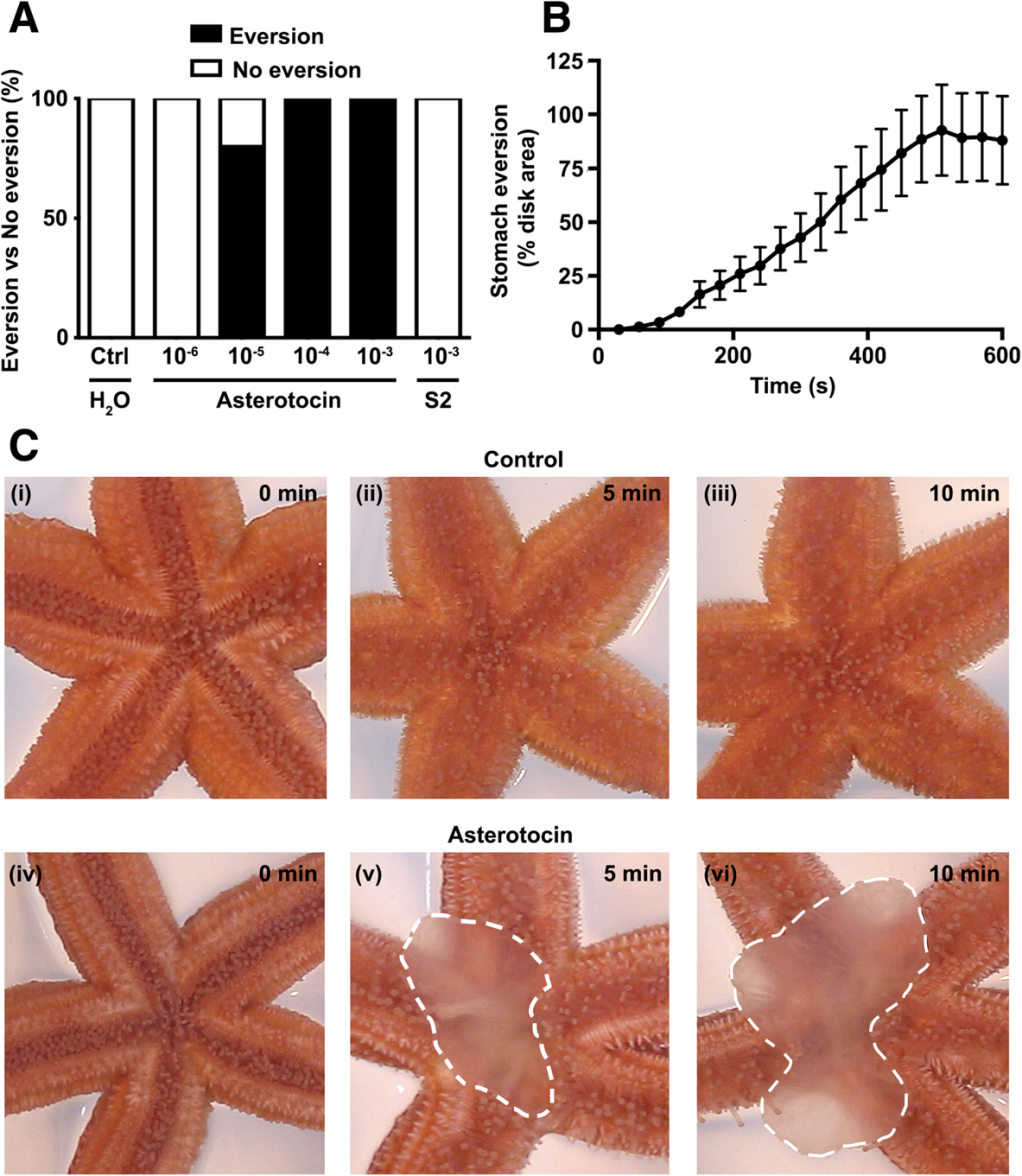
In vivo injection of asterotocin triggers cardiac stomach eversion in *A. rubens*. **a** Dose-dependent effect of asterotocin in inducing cardiac stomach eversion. The graph shows the percentage of animals (*n* = 5 per group) that exhibit cardiac stomach eversion when injected with 10 μl asterotocin at concentrations ranging from 10^−6^ M to 10^−3^ M, by comparison with 10 μl of the starfish neuropeptide S2 (10^−3^ M) or 10 μl of water. Stomach eversion occurred in 100%, 100% and 80% of the animals injected with 10^−5^ M, 10^−4^ M and 10^−3^ M asterotocin, respectively, but stomach eversion was not observed in any of the animals injected with 10^−6^ M asterotocin, 10^−3^ M S2 or water. **b** Temporal dynamics of asterotocin-induced (10 μl of 10^−3^ M) cardiac stomach eversion. The graph shows mean area (± SEM; *n* = 13) of the cardiac stomach everted expressed as the percentage of the area of the central disc region at 30-s intervals over a 10-min period following injection of asterotocin. **c** Images from video recordings of the experiment in **b** showing a representative water-injected (control) starfish (i–iii) and a representative asterotocin-injected starfish (iv–vi) at 0 min (immediately after injection), after 5 min and after 10 min. The area of the cardiac stomach everted is shown with a dashed line in v and vi. The representative video recordings used to generate the images in **c** are in Additional file 7

Starfish feed by firstly prying apart the bivalve shells of prey (e.g. mussels) with their tube feet and then everting their cardiac stomach out of their mouth and over the exposed digestible soft tissues [35, 36]. In order for the cardiac stomach to be everted, it must be in a relaxed state, and therefore, neuropeptides that cause cardiac stomach relaxation in vitro are potential mediators of eversion in vivo. We have previously reported that injection of 100 μl 10^−3^ M S2 into the perivisceral coelom of *A. rubens* triggers cardiac stomach eversion [34], and here we investigated if asterotocin also triggers cardiac stomach eversion in this species. Because the relaxing effect of asterotocin on the cardiac stomach in vitro was consistently observed at concentrations ranging from 10^−9^ to 10^−6^ M (Fig. 7b), experiments were designed to test asterotocin over a similar concentration range in vivo. Informed by analysis of the volume of the pervisceral coelomic fluid in *A. rubens* (~ 5–15 ml for animals used in this study), 10 μl of 10^−6^–10^−3^ M asterotocin (*n* = 5 for each concentration) was injected into the perivisceral coelom of starfish to achieve an estimated concentration of 10^−9^ to 10^−6^ M in vivo. For comparison, other starfish were injected with 10 μl water (*n* = 5) or 10 μl 10^−3^ M S2 (*n* = 5). No stomach eversion was observed in animals injected with water, whereas cardiac stomach eversion occurred in 0%, 80%, 100% and 100% of the animals injected with 10 μl 10^−6^–10^−3^ M asterotocin (Fig. 8a). Interestingly, cardiac stomach eversion was not observed in animals injected with 10 μl of 10^−3^ M S2, consistent with its lower potency/efficacy as a cardiac stomach relaxant in vitro by comparison with asterotocin (Fig. 7c).

To investigate more specifically the dynamics of the in vivo effect of asterotocin, we performed experiments where the effect of injection of 10 μl 10^−3^ M asterotocin was video recorded, quantifying cardiac stomach eversion as a percentage of the area of the central disc region at 30 s intervals over a period of 10 min from the time of injection. In this experiment, cardiac stomach eversion was observed in 13 out of 15 animals tested, with the remaining 2 animals displaying mouth opening but no eversion. Quantification of responses in the 13 animals where asterotocin-induced cardiac stomach eversion was observed (Fig. 8b) revealed that eversion typically started at approximately 2 min and 30 s after the injection and mean maximal eversion occurred by 8 min after the injection of asterotocin. Video recordings of representative water-injected (control) and asterotocin-injected animals are shown in Additional file 7, and images captured from these videos are shown in Fig. 8c.

### Asterotocin causes changes in body posture that affect righting behaviour

**Fig. 9:**
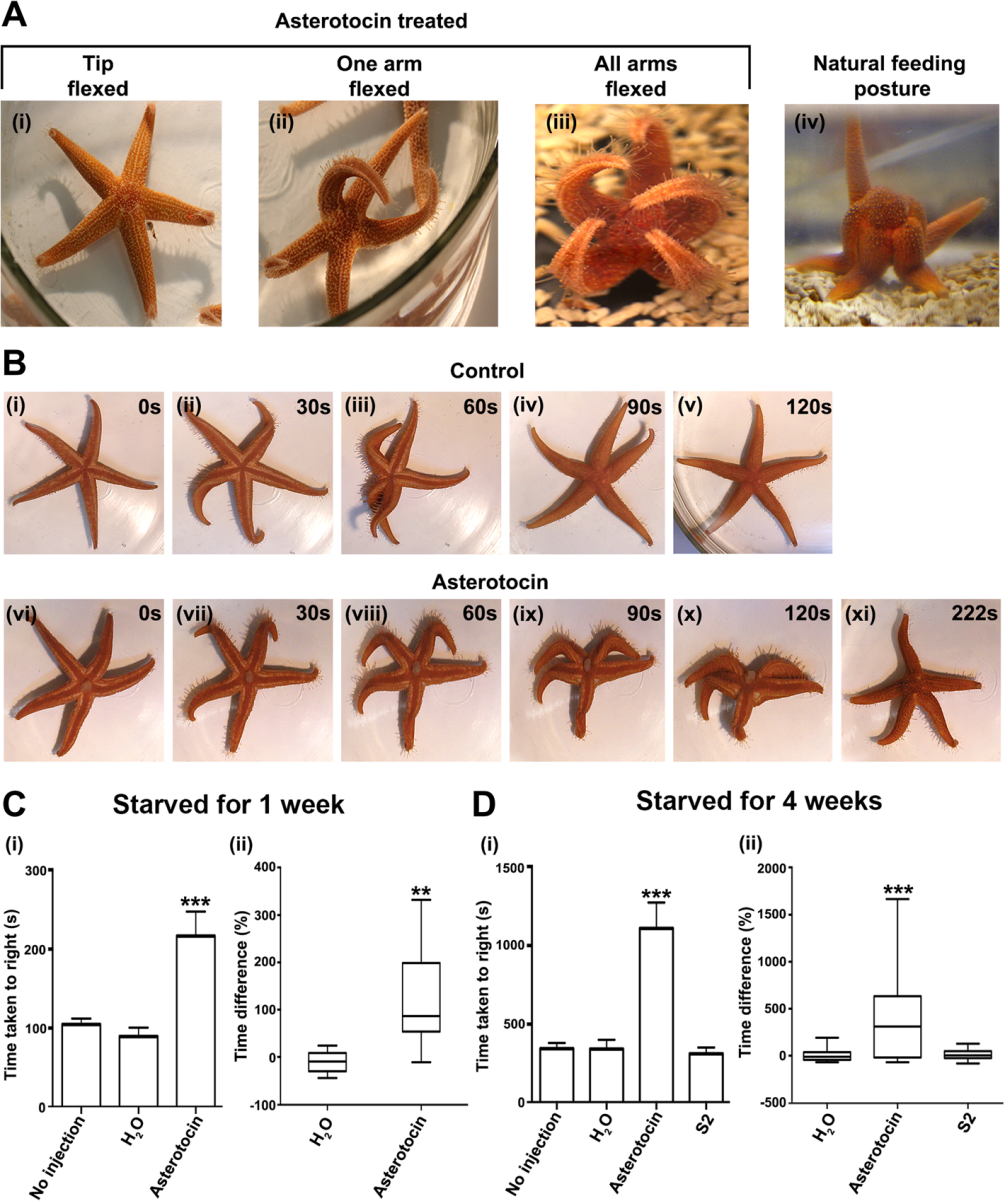
Asterotocin induces a feeding-like posture that impairs righting behaviour in *A. rubens*. **a** Asterotocin-induced changes in posture in *A. rubens*: (i) soon after injection, with arm tips curled upwards; (ii) within 10 min, with one or more arms curled upwards; and (iii) within 20 min, with all arms curled upwards and resembling the natural feeding posture (iv). **b** Images from representative videos show that asterotocin-injected starfish (vi–xi) take longer to right than water-injected starfish (i–v). **c** Effect of asterotocin on righting behaviour following 1-week starvation. (i) Mean (± SEM) righting time in asterotocin-injected starfish is 217 ± 31 s (*n* = 10) and significantly longer than in non-injected (105 ± 8 s; *n* = 20, pooled data) and water-injected starfish (89 ± 11 s; *n* = 10); (*P* < 0.0001; one-way ANOVA with Dunnett’s multiple comparisons test). (ii) Mean percentage righting time difference between water-injected and non-injected starfish is − 11 ± 7%, whereas between asterotocin-injected and non-injected starfish, it is 127 ± 36% (*P* = 0.0005; Mann-Whitney *U* test; *n* = 10). **d** Testing effects of asterotocin and S2 on righting behaviour following 4-week starvation. (i) Mean (± SEM) righting times in water-injected animals (341 ± 59 s; *n* = 20) and in S2-injected animals (312 ± 40 s; *n* = 20) are significantly different. Asterotocin causes a significant increase in righting time (1110 ± 162 s; *n* = 20) compared with non-injected (*n* = 60, pooled from the three treated groups), water-injected and S2-injected animals (*P* < 0.0001; one-way ANOVA with Dunnett’s multiple comparisons test). (ii) Mean percentage righting time difference between non-injected and water-injected animals (− 1.8 ± 13%) and between non-injected and S2-injected animals (15 ± 12%) are not statistically significant, but there is statistical significance between non-injected and asterotocin-injected animals (414 ± 104%; *P* < 0.0001; one-way ANOVA with Dunnett’s multiple comparisons test; *n* = 20)

While investigating the effect of asterotocin in triggering cardiac stomach eversion in *A. rubens*, we observed that arm flexion occurred in many of the animals injected with asterotocin but not in control animals injected with water. The magnitude of this effect was variable, however, with flexion of one or more arm tips occurring (Fig. 9a (i)), flexion of one or two whole arms occurring (Fig. 9a (ii)) or flexion of all five arms occurring (Fig. 9a (iii)). This is interesting because, relevant to the effect of asterotocin in triggering cardiac stomach eversion, arm flexion occurs in starfish when they adopt a humped feeding posture to prey on mussels and other marine invertebrates [36] (Fig. 9a (iv)). To assess the impact of the effect of asterotocin on body posture in *A. rubens*, we tested the effect of asterotocin on the righting behaviour of starfish. Righting occurs if starfish are upturned so that their oral surface is uppermost, with animals twisting their arms so that the tube feet can latch onto the substratum and then somersaulting to bring the entire oral surface back into contact with the substratum [37, 38]. Following a 1-week period of starvation to normalise the physiological condition of animals, the effect of asterotocin injection and water injection on starfish righting behaviour was compared. A representative experiment is shown in Fig. 9b, where it can be seen that the water-injected animal has righted within 120 s, whereas by this time point, the asterotocin-injected animal still has its oral surface uppermost and cardiac stomach eversion can also be seen. Righting in the asterotocin-injected animal is not completed until 222 s after injection. Analysis of data obtained from all of the animals tested in this experiment revealed that the mean time taken for righting to occur in water-injected starfish was 89 ± 11 s whereas the time taken for righting to occur in animals injected with asterotocin was significantly longer, with a mean of 217 ± 31 s (Fig. 9c (i)). Because of inter-individual variation in the time taken for righting to occur, the percentage mean difference in righting time with and without injection of water or asterotocin was also calculated (Fig. 9c (ii)). The mean percentage righting time difference between water-injected and non-injected starfish was − 11 ± 7%, while asterotocin-injected animals exhibited a 127 ± 36% increase in the righting time.

In a separate experiment, the effect of asterotocin on righting behaviour was investigated by comparison with S2, a neuropeptide which like asterotocin induces cardiac stomach eversion but which does not induce the changes in posture seen in animals injected with asterotocin (Fig. 9d). For this experiment, the animals were starved for a longer period (4 weeks), and the mean time for righting to occur in non-injected animals was longer (345 ± 35 s) compared to the previous experiment (105 ± 8 s), which probably reflects the longer starvation period prior to testing. The mean righting time for S2-injected animals (312 ± 40 s) was not significantly different to the mean righting time of water-injected animals (341 ± 59 s), whereas the mean righting time of asterotocin-injected animals was significantly longer (1110 ± 162 s) (Fig. 9d (i)). Furthermore, by comparing the righting time before and after the injection, the mean percentage differences following the injection of water and S2 were not statistically significant: − 1.8 ± 13% and 15 ± 12%, respectively. In contrast, the mean percentage righting time difference following injection of asterotocin was 414 ± 104% (Fig. 9d (ii)). In conclusion, these experiments indicate that the effect of asterotocin on starfish righting behaviour is attributable to its effect on posture.

## Discussion

Investigation of the actions of VP/OT-type neuropeptides in chordates, and protostomian invertebrates has revealed conserved and evolutionarily ancient roles in the regulation of processes such as reproduction and water homeostasis [10–12]. To obtain new insights into the evolution of VP/OT-type neuropeptide function in the animal kingdom, here we functionally characterised VP/OT-type signalling in an echinoderm—the starfish *A. rubens*. Investigation of VP/OT-type neuropeptide function in echinoderms is of special interest because they are deuterostomian invertebrates and therefore are more closely related to chordates than protostomes. Furthermore, the derived radially symmetrical body plan of adult echinoderms provides a unique context for the investigation of neuropeptide function in the Bilateria [39].

### The vasopressin/oyxtocin-type neuropeptide asterotocin acts as a muscle relaxant and triggers fictive feeding in starfish

Investigation of the in vitro pharmacological actions of the VP/OT-type neuropeptide asterotocin in starfish revealed an unusual functional characteristic in that it acts as a muscle relaxant, whereas in other taxa, VP/OT-type neuropeptides typically cause muscle contraction [15, 18, 40–44]. More specifically, we discovered that asterotocin is a potent relaxant of in vitro preparations of the cardiac stomach from starfish. This was of interest from a physiological/behavioural perspective because relaxation of the cardiac stomach occurs naturally when starfish evert their stomach out of their mouth to feed extra-orally on prey (e.g. mussels) [35, 36]. Previous studies have revealed that neuropeptides belonging to the echinoderm SALMFamide neuropeptide family also act as cardiac stomach relaxants in vitro and can trigger cardiac stomach eversion when injected in vivo [33, 34]. Therefore, here we compared the effects of in vivo injection of asterotocin and the SALMFamide neuropeptide S2. This revealed that asterotocin also triggers cardiac stomach eversion in starfish, but it is much more effective than S2. Furthermore, the observation of starfish that had been injected with asterotocin revealed that it also induced postural changes resembling the humped posture that starfish adopt when feeding on prey [36]. Strikingly, this effect of asterotocin on starfish body posture was so powerful that it prevented upturned starfish from righting themselves normally. Thus, injection of asterotocin induces fictive feeding in starfish, triggering both cardiac stomach eversion and adoption of a body posture resembling that which occurs during natural feeding on prey. Based on these findings, we propose that the VP/OT-type neuropeptide asterotocin has a physiological role as a neural regulator of the unusual extra-oral feeding behaviour of starfish.

### Mechanisms of asterotocin signalling in starfish

To gain insights into the molecular and cellular mechanisms of asterotocin signalling in starfish, we identified an *A. rubens* G-protein coupled receptor which is an ortholog of VP/OT-type receptors that have been characterised pharmacologically in other taxa. Furthermore, we discovered that asterotocin acts as a potent ligand (EC_50_ = 5.7 × 10^−8^ M) for the *A. rubens* VP/OT-type receptor when the receptor is heterologously expressed in CHO cells. Thus, the molecular components of a VP/OT-type neuropeptide signalling system, asterotocin and a G-protein coupled asterotocin receptor, were identified biochemically in *A. rubens*. Importantly, this enabled analysis of the distribution of molecular components of the VP/OT-type signalling pathway in *A. rubens*, providing an anatomical framework for interpretation of the in vitro and in vivo effects of asterotocin in this species. Furthermore, it is noteworthy that this is the first study to determine the anatomical expression patterns of both a neuropeptide and its cognate receptor in an echinoderm.

Consistent with the in vitro and in vivo effects of asterotocin in triggering cardiac stomach relaxation and eversion in *A. rubens*, respectively, cells expressing the asterotocin precursor transcript were detected in the mucosal layer of the cardiac stomach, and asterotocin immunoreactivity was revealed in the basiepithelial nerve plexus of the cardiac stomach. Accordingly, cells expressing the asterotocin receptor transcript were detected in the mucosal and visceral muscle layers of the cardiac stomach, and asterotocin receptor immunoreactivity was revealed both in the basiepithelial nerve plexus and in the visceral muscle layer of the cardiac stomach. Based on these expression patterns and the pharmacological effects of asterotocin in *A. rubens*, it can be inferred that asterotocin is released physiologically by neurons located in the mucosal layer of the cardiac stomach and then (i) diffuses into the visceral muscle layer to act on asterotocin receptors on muscle cells to cause relaxation and/or (ii) acts on neural processes in the basiepithelial nerve plexus layer to trigger release of another neurochemical that acts directly on muscle cells to cause relaxation.

The effect of asterotocin in inducing a posture that resembles the humped feeding posture of starfish is interesting because this effect has not been reported for any other neuropeptide. It also raises questions regarding the mechanisms by which asterotocin exerts this effect. Our analysis of the distribution of asterotocin in the central nervous system of *A. rubens* revealed that the asterotocin precursor transcript and asterotocin immunoreactivity are restricted to the ectoneural regions of the radial nerve cords and circumoral nerve ring, with no expression detected in the hyponeural region. This is noteworthy because it is the hyponeural region that contains the cell bodies and processes of skeletal motoneurons [32, 45, 46]. In starfish, the skeleton comprises a network of calcite ossicles that are linked by interossicular muscles, which enable changes in body posture [47]. Consistent with the absence of asterotocin expression in neuronal cell bodies of hyponeural motoneurons, no asterotocin immunoreactivity was observed in the innervation of the interossicular muscles. This contrasts with other neuropeptides in starfish, such as pedal peptide-type neuropeptides and a calcitonin-type neuropeptide, which are expressed by hyponeural neuronal cell bodies and are present in the innervation of interossicular muscles [48–50]. Like asterotocin, both pedal peptide-type and calcitonin-type neuropeptides act as muscle relaxants in starfish, but unlike asterotocin, they do not trigger cardiac stomach eversion or a feeding posture [48–50]. We infer from these observations that asterotocin may induce a feeding posture in starfish by acting within the ectoneural region of the central nervous system to cause activation of downstream asterotocin receptor-mediated neural mechanisms that induce postural changes.

The distribution of asterotocin indicates that it is not solely involved in the regulation of feeding behaviour in starfish. For example, asterotocin and its receptor are detected in the body wall and its associated appendages, which is suggestive of a role in mediating local responses to the sensory stimuli. Additionally, asterotocin is detected in cells/processes associated with the apical muscle, which is innervated by cells located in the coelomic epithelium that lines the inner surface of the aboral body wall, and consistent with this pattern of expression, asterotocin causes relaxation of apical muscle preparations in vitro. Thus, asterotocin is not dissimilar to VP/OT-type neuropeptides that have been functionally characterised in other taxa, where pleiotropic actions are indicative of roles in the regulation of a variety of physiological/behavioural processes [16, 29, 51]. It will be of interest to examine other aspects of asterotocin function in starfish in future studies, and not only in adult animals but also in the free-swimming and bilaterally symmetrical starfish larvae. Preliminary insights have been obtained by mapping the distribution of the asterotocin precursor transcript in the larvae of *A. rubens*, which revealed expression in cells associated with the attachment complex that mediates larval attachment to the substratum prior to metamorphosis [22].

### Evolution and comparative physiology of VP/OT-type neuropeptides as regulators of feeding

Our discovery that asterotocin has such striking feeding-related effects in starfish provides a basis to examine if VP/OT-type neuropeptides are likewise involved in the regulation of feeding-associated processes in other taxa. Currently, little is known about the physiological roles of VP/OT-type neuropeptides in other echinoderms. However, it has been reported that a VP/OT-type neuropeptide (echinotocin) causes contraction of in vitro preparations of the oesophagus and tube feet in sea urchins [18]. This effect of echinotocin as a muscle contractant is consistent with the myoexcitatory effects of VP/OT-type neuropeptides in many other taxa, but it contrasts with the relaxing action of asterotocin on starfish muscle preparations. Thus, this highlights how unusual the myoinhibitory action of asterotocin is as a VP/OT-type neuropeptide in starfish. Nevertheless, the effect of echinotocin on the sea urchin oesophagus suggests that VP/OT-type neuropeptides may be generally associated with the regulation of feeding-related processes in echinoderms. Turning to other deuterostomian invertebrates, currently, nothing is known about the physiological roles of the VP/OT-type neuropeptides that been identified by analysis of genome sequence data in hemichordates and cephalochordates. However, a VP/OT-type signalling system has been characterised in the urochordate *Ciona intestinalis*, revealing that the VP/OT-type receptor Ci-VP-R is expressed in the alimentary tract [27]. In vertebrates, VP/OT-type neuropeptides are, as highlighted above, most widely known for their effects on reproductive physiology/behaviour and osmoregulation [4, 5]. However, roles in the regulation of feeding and/or digestive processes have been reported. Thus, there is a substantial body of evidence that OT inhibits feeding [52–55] and gastric motility [56, 57] in mammals. Accordingly, the OT-type peptide isotocin and the VP-type peptide vasotocin inhibit food intake in fish [58, 59]. Although the effect of asterotocin in inducing fictive feeding in starfish contrasts with the inhibitory actions of VP/OT-type neuropeptides on feeding in vertebrates, our findings indicate that VP/OT-type neuropeptides are evolutionarily ancient regulators of feeding-related processes in the deuterostome branch of the Bilateria.

What about in the protostome branch of the Bilateria? In the annelid *Eisenia foetida*, the VP/OT-type neuropeptide annetocin potentiates spontaneous contractions of gut preparations, providing evidence of a role in the regulation of digestive processes [60]. In the cephalopod mollusc *Octopus vulgaris*, the VP/OT-type signalling system has been characterised in detail and comprises two VP/OT-type neuropeptides, octopressin (OP) and cephalotocin (CT), and three cognate receptors (OPR, CTR1, CTR2). Furthermore, OP is expressed in the regions of the octopus nervous system involved in the control of feeding and/or gut activity and, accordingly, OP has myoexcitatory effects on in vitro preparations of the octopus rectum [42, 61, 62]. However, experimental evidence that OP regulates feeding behaviour in octopuses has as yet not been reported. Interestingly, extensive analysis of VP/OT-type signalling in the nematode *C. elegans* has revealed that, in addition to a reproductive role in the regulation of mating behaviour [16], VP/OT-type signalling regulates gustatory associative learning in this species [29]. Thus, both direct and indirect evidence that VP/OT-type signalling is involved in the regulation of feeding-related processes have been obtained from a variety of studies on protostomes.

We infer from this phylogenetic survey of VP/OT-type neuropeptide function that the effect of asterotocin in triggering fictive feeding in starfish reflects an evolutionarily ancient role of VP/OT-type neuropeptides as regulators of feeding-related processes in the Bilateria. This adds to the existing evidence of evolutionarily ancient roles in the regulation of diuresis and reproductive processes and reflects the pleitropy of neuropeptide function in animals. However, the extent to which evolutionarily ancient roles of VP/OT-type neuropeptides as regulators of feeding, reproduction and diuresis have been preserved in different taxa may vary considerably. Therefore, further investigation of VP/OT-type neuropeptide function in a variety of bilterian phyla and classes will be needed to gain deeper insights into the evolution of the physiological roles of this signalling system.

## Conclusions

Here, we have reported a comprehensive characterisation of VP/OT-type signalling in an echinoderm, the starfish *A. rubens*. This has yielded striking evidence that the VP/OT-type signalling system may be a key regulator of the unusual extra-oral feeding behaviour of starfish. Furthermore, when combined with previous reports of feeding-related roles in vertebrates and protostomes, our findings provide important new evidence that VP/OT-type neuropeptide signalling is an ancient and evolutionarily conserved regulator of feeding in the Bilateria.

## Materials and methods

### Animals

Starfish (*A. rubens*) were collected from the Thanet Coast (Kent, UK) at low tide or were acquired from a fisherman based in Whitstable (Kent, UK) and transported to the School of Biological and Chemical Sciences, Queen Mary University of London. The starfish were maintained in circulating artificial seawater at approximately 12 °C in an aquarium and were fed mussels (*Mytilus edulis*). Animals ranging in diameter from 5 to 15 cm were used for in vitro and in vivo pharmacological experiments, whereas smaller animals (< 5 cm in diameter) were used for anatomical studies. Additionally, juvenile specimens of *A. rubens* (diameter 0.5–1.5 cm) were collected from the University of Gothenburg Sven Lovén Centre for Marine Infrastructure (Kristineberg, Sweden) and used for anatomical studies.

### Mass spectrometric identification of the VP/OT-type neuropeptide asterotocin in *A. rubens* radial nerve cord extracts

Radial nerve cords were dissected from five adult specimens of *A. rubens* using a method described previously [63]. Neuropeptides were then extracted in 1 ml of 80% acetone on ice [64]. Acetone was removed by evaporation using nitrogen, with the aqueous fraction centrifuged at 11,300*g* in a MiniSpin® microcentrifuge (Eppendorf) for 10 min, and the remaining supernatant stored at − 80 °C. Prior to mass spectrometry (MS), the extract was thawed (with an aliquot diluted tenfold with 0.1% aqueous formic acid) and filtered through a 0.22-μm Costar Spin-X centrifuge tube filter (Sigma-Aldrich) to remove particulates. In comparison with synthetic asterotocin (PPR Ltd., Fareham, UK), the radial nerve cord extract was analysed by nanoflow liquid chromatography (LC) with electrospray ionisation (ESI) quadrupole time-of-flight tandem MS (nano LC-ESI-MS/MS) using a nanoAcquity ultra performance LC (UPLC) system coupled to a Synapt® G2 High-Definition Mass Spectrometer™ (HDMS) (Waters Corporation) and MassLynx v4.1 SCN 908 software (Waters Corporation). The mobile phases used for the chromatographic separation were 0.1% aqueous formic acid (termed mobile phase A) and 0.1% formic acid in acetonitrile (termed mobile phase B). An aliquot containing 15 μl of the *A. rubens* radial nerve cord extract was applied to a Symmetry C18® (180 μm × 20 mm, 5 μm particle size, 100 Å pore size) trapping column (Waters Corporation) using 99.9% mobile phase A at a flow rate of 10 μl min^−1^ for 3 min, after which the fluidic flow path included the HSS T3 (75 μm × 150 mm, 1.8 μm particle size, 100 Å pore size) analytical capillary column (Waters Corporation). A linear gradient of 5–40% mobile phase B over 105 min was utilised with a total run time of 120 min. The nanoflow ESI source conditions utilised a 3.5-kV capillary voltage, 25 V sample cone voltage and an 80 °C source temperature. The instrument was operated in resolution mode (~ 20,000 measured at full width and half height). A solution containing 500 fmol μl^−1^ Glu^1^-Fibrinopeptide B peptide in 50% *v*/*v* aqueous acetonitrile containing 0.1% formic acid was infused via a NanoLockSpray interface at a constant rate of 500 nl min^−1^, sampled every 60 s and used for lockmass correction (m/z 785.8426) enabling accurate mass determination.

A data-dependent acquisition was performed that would trigger an MS/MS scan on any singly charged peptide having a mass to charge ratio (m/z) of 960.3919, or a doubly charged peptide of m/z 480.6999, with a tolerance of 100 mDa allowed on the precursor m/z. MS/MS spectra, obtained from data-dependent acquisition, were processed using MassLynx™ software (Waters Corporation). Spectra were combined and processed using the MaxEnt 3 algorithm to generate singly charged, monoisotopic spectra for interpretation and manual validation.

### Identification and cloning of a cDNA encoding an *A. rubens* VP/OT-type receptor

To identify an *A. rubens* VP/OT-type receptor, the amino acid sequence of the sea urchin (*Strongylocentrotus purpuratus*) VP/OT-type receptor [65] was submitted as a query in a tBLASTn search of *A. rubens* radial nerve cord transcriptome sequence data using SequenceServer software [66]. The top hit (contig 1122053) was a 2710-bp transcript encoding a 428-residue protein, which based on reciprocal BLAST analysis was identified as an ortholog of the *S. purpuratus* VP/OT-type receptor. A cDNA encoding the *A. rubens* VP/OT-type receptor was then cloned using *A. rubens* radial nerve cord cDNA as a template for PCR amplification, employing the use of primers (see Additional file 2) that were designed using Primer3 (http://primer3.ut.ee). The cDNA was then incorporated into the pBluescript SKII (+) vector and sequenced (Eurofins Genomics).

### Phylogenetic analysis of the *A. rubens* VP/OT-type receptor

Phylogenetic analysis of the relationships between the *A. rubens* VP/OT-type receptor and VP/OT-type receptors from other species was performed using the maximum-likelihood method. Firstly, receptor protein sequences were aligned in MEGA7 (v.7170509) using MUltiple Sequence Comparison by Log-Expectation (MUSCLE) [67]. Once aligned, poorly aligned regions were removed using the Gblocks server (using the least stringent settings) to optimise the alignment for phylogenetic analysis [68]. PhyML (version 3.0) was then used to generate a maximum-likelihood tree [69]. The LG model was automatically selected, and the bootstrap was manually set to 1000. FigTree version 1.4.3 (http://tree.bio.ed.ac.uk/software/figtree/) was used to visualise and re-root the tree generated by PhyML. NPS/CCAP/NG peptide-type, and GnRH/AKH/ACP/CRZ-type receptor sequences were used as outgroups in the tree.

### Testing asterotocin as a ligand for the *A. rubens* VP/OT-type receptor

A pBluescript SKII (+) vector containing the *A. rubens* VP/OT-type receptor cDNA as an insert (see above) was used as a template to amplify by PCR the open reading frame of the VP/OT-type receptor. To accomplish this, the oligonucleotides 5′-ggatccCACCATGACGCCCTC-3′ (upstream) and 5′-cccgggCTACATGTGAGCGGAAGCA-3′ (downstream) were used as primers and the PCR product was subcloned into the eukaryotic expression vector pcDNA 3.1+, which had been cut with the restriction enzymes BamHI and ApaI. For the upstream primer, a partial Kozak translation initiation sequence (CACC) was introduced before the start codon to optimise the initiation of translation.

To determine if asterotocin acts as a ligand for the *A. rubens* VP/OT-type receptor, a cell-based assay was used where Ca^2+^-induced luminescence is measured. For this assay, Chinese hamster ovary (CHO-K1) cells stably expressing the Ca^2+^-sensitive bioluminescent GFP-aequorin fusion protein—G5A (CHO-K1/G5A cells [70]) are co-transfected with plasmids containing a G-protein coupled receptor cDNA insert and plasmids containing an insert encoding the promiscuous G-protein Gα16. If a candidate ligand activates the expressed receptor, the promiscuous Gα16 protein triggers the activation of the IP_3_ signalling pathway, causing an increase in intracellular Ca^2+^ and luminescence. We recently reported the use of this assay to demonstrate that the luqin-type neuropeptide ArLQ acts as a ligand for the *A. rubens* G-protein coupled receptors ArLQR1 and ArLQR2 [71], and therefore, here only a brief outline of methods employed is described. CHO-K1/G5A cells were transfected with 5 μg of the pcDNA 3.1+ plasmid containing the *A. rubens* VP/OT-type receptor cDNA and 1.5 μg of plasmid containing an insert encoding the promiscuous Gα16 subunit using the Lipofectamine 3000® Transfection Kit, (Invitrogen). The cells were then loaded with the aqueorin substrate coelenterazine-H (Thermo Fisher Scientific). Asterotocin at concentrations ranging from 10^−4^ to 10^−12^ M (*n* = 3) was pipetted into the wells of clear-bottomed 96-well plates (Sigma-Aldrich), then a FLUOstar Omega Plate Reader (BMG LABTECH) was used to inject a fixed amount of transfected CHO-K1 cells into each well sequentially, and luminescence was measured for a 35-s period after injection. Luminescence measurements were normalised to the maximum response obtained in each experiment (100%) and the response obtained with the vehicle media (0%). These data (three repeats per experiment and at least three independent transfections) were used to construct a dose-response curve utilising non-linear regression analysis in Prism 6.0c (GraphPad, La Jolla, USA) and displayed on a semi-logarithmic plot. The half maximal effective concentration (EC_50_) was calculated from the dose-response curve in Prism 6.0c. For negative control experiments, CHO-K1/G5A cells were transfected with the empty pcDNA 3.1+ vector. Human vasopressin and oxytocin and the *A. rubens* neuropeptide NGFFYamide were also tested to assess the specificity of asterotocin as the candidate ligand for the *A. rubens* VP/OT-type receptor. A Wilcoxon signed-rank test was performed to compare luminescence of cells exposed to asterotocin, vasopressin, oxytocin and NGFFYamide (at 10^−4^ M) with luminescence of cells in basal media (control).

### Localisation of the expression asterotocin precursor and asterotocin receptor transcripts in *A. rubens* using in situ hybridisation

The method employed for the production of the asterotocin precursor antisense and sense digoxygenin (DIG)-labelled RNA probes is as described in [22]. Production of the asterotocin receptor antisense and sense DIG-labelled RNA probes was performed as follows. The plasmid containing the cloned asterotocin receptor cDNA was linearised using the restriction enzymes HindIII and EcoRI (NEB, Hitchin, Hertfordshire, UK). Once linearised, the plasmids were purified using phenol-chloroform/chloroform isomylalcohol (Sigma-Aldrich Ltd., Gillingham, UK) extraction. RNA probes were synthesised from the purified, linearised plasmid using a DIG-labelled nucleotide triphosphate mix (Roche, Nutley, NJ) supplemented with dithiothreitol (Promega), a placental RNase inhibitor (Promega), and RNA polymerases (New England Biolabs), according to the manufacturer’s instructions. To synthesise the antisense and sense probes, T3 and T7 RNA polymerases were used, respectively. Reaction products were digested with RNase-free DNase (New England Biolabs) to remove template DNA and then stored in 25% formamide made up in 2× saline-sodium citrate (SSC) buffer at − 20 °C.

The methods employed for preparation of sections of 4% paraformaldehyde-fixed specimens of *A. rubens* and visualisation of asterotocin precursor and asterotocin receptor transcripts in these sections were the same as those used previously for the analysis of AruRGPP expression, as reported in [72]. For the visualisation of asterotocin precursor transcripts, the sections were incubated with antisense and sense RNA probes at a concentration of 800 ng/ml. Then, following incubation with anti-DIG antibodies, slides were incubated with the staining solution at room temperature for a few hours until strong staining was observed. For the visualisation of asterotocin receptor transcripts, the sections were incubated with antisense and sense RNA probes at a concentration of 1500 ng/ml. Then, following incubation with anti-DIG antibodies, the slides were incubated with the staining solution at room temperature for a few hours, left overnight at 4 °C and then were incubated at room temperature for several more hours until strong staining was observed.

### Production, characterisation and purification of rabbit antibodies to asterotocin and guinea pig antibodies to the asterotocin receptor

To generate antibodies to asterotocin, a rabbit was immunised with a conjugate of thyroglobulin (carrier protein) and the peptide Lys-asterotocin (KCLVQDCPEG-NH_2_; disulphide bridge between the cysteine residues), with the N-terminal lysine providing a free amine group for glutaraldehyde-mediated coupling to thyroglobulin. To generate antibodies to the asterotocin receptor, a guinea pig was immunised with a conjugate of thyroglobulin and a peptide corresponding to the C-terminal region of the asterotocin receptor sequence (KFVSTTGTASAHM) but with an additional N-terminal lysine providing a free amine group for glutaraldehyde-mediated coupling to thyroglobulin.

The peptide antigens were synthesised by Peptide Protein Research Ltd. (Fareham, Hampshire, UK). Conjugation of the antigen peptides to thyroglobulin was performed as described in [20]. Rabbit immunisation and serum collection were performed by Charles River Biologics (Romans, France) according to the following protocol. On day 0, pre-immune serum was collected and the first immunisation (~ 100 nmol of conjugated antigen peptide emulsified in Freund’s complete adjuvant) was administered. Booster immunisations (~ 50 nmol of conjugated antigen peptide emulsified in Freund’s incomplete adjuvant) were administered on days 28, 42 and 56. Samples of blood serum were collected on days 38 and 52, and the final bleed serum was collected on day 70. Guinea pig immunisation and serum collection were performed by Charles River Biologics (Romans, France) according to the following protocol. On day 0, pre-immune serum was collected and the first immunisation (~ 100 nmol of conjugated antigen peptide emulsified in Freund’s complete adjuvant) was administered. Booster immunisations (~ 50 nmol of conjugated antigen peptide emulsified in Freund’s incomplete adjuvant) were administered on days 14, 28 and 42. A serum sample was collected on day 38, and the final bleed serum was collected on day 56.

Enzyme-linked immunosorbent assays (ELISA) were performed to test the sera for the presence of antibodies to the antigen peptides, employing the same methods as described previously for ArGnRH antisera [20]. Terminal bleed antisera were characterised by ELISA by testing antisera at a starting dilution of 1:500 and subsequent twofold serial dilutions down to 1:16,000 (Additional file 4). Then, antibodies to the antigen peptides were affinity-purified from terminal bleed antisera using AminoLink® Plus Immobilisation Kit (Thermo Scientific), employing the same methods as described previously for antibodies to ArGnRH [20].

### Localisation of the expression of asterotocin and the asterotocin receptor in *A. rubens* using immunohistochemistry

The methods employed for preparation of sections of Bouin’s fixed specimens *A. rubens* and immunohistochemical localisation of asterotocin expression and asterotocin receptor expression were the same as those described previously for ArPPLN1 and ArGnRH [21, 48]. The sections were incubated overnight or for 3 days, respectively, with affinity-purified rabbit antibodies to asterotocin (1:4 dilution) and guinea pig antibodies to the asterotocin receptor (1:4 dilution). Then, bound antibodies were visualised using diaminobenzidine as a substrate for peroxidase-conjugated AffiniPure Goat anti-rabbit immunoglobulins (Jackson ImmunoResearch Laboratories, West Grove, PA, USA) or peroxidase-conjugated AffiniPure Donkey anti-guinea pig immunoglobulins (Jackson ImmunoResearch Laboratories, West Grove, PA, USA).

To visualise asterotocin and the asterotocin receptor in the sections using double-labelling fluorescence immunohistochemistry, the sections were first incubated with affinity-purified asterotocin antibodies (1:4 dilution) overnight at 4 °C. Then, following washes (4 × 5 min) in phosphate-buffered saline (PBS) containing 0.1% Tween-20 (PBST) and washes in PBS (4 × 10 min), the sections were incubated with affinity-purified antibodies to the asterotocin receptor (1:4 dilution) for 2 weeks at 4 °C. Then, following washes with PBST and PBS (as described above), the sections were incubated for 3 to 4 h with Cy2-labelled goat anti-rabbit immunoglobulins and Cy3-labelled goat anti-guinea pig immunoglobulins (Jackson ImmunoResearch Europe Ltd., UK), which were both diluted 1:200 in 2% normal goat serum in PBS. Following washes in PBST and PBS (as described above), the slides were mounted with coverslips using Fluoroshield Mounting Medium with DAPI (Abcam, Cambridge, UK).

To capture images of sections labelled using fluorescence immunohistochemistry, a Leica SP5 confocal microscope was used in combination with the Leica Application Suite Advanced Fluorescence (LAS AF; version 2.6.0.7266) programme. Argon and DPSS 561 lasers were used for the detection of green fluorescence (asterotocin) and red fluorescence (asterotocin receptor), respectively. For Beam Path Settings, FITC and TRITC were selected, and in the case of TRITC, the PMT was set to Cy3. While visualising immunofluorescence on slides, the smart gain, smart offset and *z* position were adjusted to generate optimal immunofluorescent images. The settings for capturing images were as follows: image format, 1024 × 1024; scan speed, 200 Hz; frame average, 6; and line average accumulation, 3. The FITC and TRITC images were taken separately, and the colour channels were merged using ImageJ to produce double-labelled images. Contrast and levels were adjusted in ImageJ, and montages were created in Adobe Photoshop CC 2017.1.1.

### Analysis of the in vitro pharmacological effects of asterotocin on cardiac stomach, apical muscle and tube foot preparations from *A. rubens*

To investigate if asterotocin affects muscle contractility in *A. rubens*, synthetic asterotocin (custom synthesised by PPR Ltd., Fareham, Hampshire, UK) was tested on three in vitro preparations: the cardiac stomach, apical muscle and tube feet. These three preparations have been used to examine the effects of other neuropeptides, and the methods employed have been described in detail previously [30, 33, 34, 73]. Preliminary tests revealed that asterotocin caused relaxation of the cardiac stomach and apical muscle preparations but had no effect on the contractile state of tube foot preparations. Therefore, the effects of asterotocin on cardiac stomach and apical muscle preparations were examined in more detail.

Cardiac stomach preparations from 16 starfish (8–13.5 cm in diameter) were incubated at 11 °C in an aerated organ bath containing artificial seawater with 3 × 10^−2^ M added KCl. This induces sustained contraction of the cardiac stomach, which facilitates recording of the effects of neuropeptides that act as muscle relaxants [34]. The contractile state of preparations was monitored using a high-grade isotonic transducer (model 60-3001; Harvard Apparatus, Cambridge, UK) connected to a Goerz SE 120 chart recorder (Recorderlab, Sutton, Surrey, UK) or using a high-grade isotonic transducer (MLT0015; ADInstruments Ltd., Oxford, UK) connected to data acquisition hardware (PowerLab 2/26, ADInstruments Ltd.) via a bridge amplifier (FE221 Bridge Amp, ADInstruments Ltd.). The output from the PowerLab was recorded using LabChart (v8.0.7) software installed on a laptop computer (Lenovo E540, Windows 7 Professional). To obtain representative images of the effects of asterotocin, traces were exported from LabChart into Adobe Photoshop and the function ‘Select and Mask’ was utilised to remove the background grid pattern.

To investigate the dose dependence and potency of asterotocin as a cardiac stomach relaxant, it was tested at concentrations ranging from 3 × 10^−11^ M to 10^−6^ M (*n* = 16). The percentage relaxation at each concentration was calculated relative to the maximal relaxing effect produced by asterotocin in each preparation. To enable the assessment of the magnitude of the relaxing effect of asterotocin on cardiac stomach, experiments were performed to compare the effect asterotocin with the effect of the neuropeptide SALMFamide-2 (S2; SGPYSFNSGLTF-NH_2_), which has been shown previously to cause relaxation of *A. rubens* cardiac stomach preparations in vitro [33, 34]. Having established that the maximal relaxing effect of asterotocin is observed at concentrations ranging from 3 × 10^−9^ M to 10^−6^ M (with a mean of ~ 10^−7^ M), experiments were performed where the effects of 10^−7^ M asterotocin and 10^−7^ M S2 on cardiac stomach preparations (*n* = 5) were compared. For these experiments, the effect of 10^−7^ M S2 was defined as 100%, and the effect of 10^−7^ M asterotocin was calculated as a percentage of the effect of 10^−7^ M S2. The relaxing effects of asterotocin and S2 on cardiac stomach preparations were analysed statistically using the Mann-Whitney *U* test.

To examine the effects of asterotocin on apical muscle preparations, 10^−6^ M acetylcholine (ACh) was used to induce contraction prior to application of asterotocin. A relaxing effect of asterotocin was observed only at a high concentration (10^−6^ M) and therefore it was not possible to examine the dose dependency of this effect. However, the experiments were performed in which the time course of the relaxing effect of asterotocin was analysed by measuring the percentage reversal of 10^−6^ M ACh-induced contraction over a 50-s period after application of 10^−6^ M asterotocin (*n* = 4).

### Analysis of the in vivo effects of asterotocin on *A. rubens*

During feeding in *A. rubens*, the cardiac stomach is everted out of the mouth over the digestible soft tissue of prey (e.g. mussels), and to accomplish this, the cardiac stomach must be relaxed. Previous studies have revealed that S2, a neuropeptide that induces relaxation of *A. rubens* cardiac stomach preparations in vitro, induces cardiac stomach eversion when injected in vivo [34]. Having established that asterotocin causes relaxation of cardiac stomach preparations in vitro, experiments were performed to investigate if asterotocin also triggers cardiac stomach eversion when injected in vivo. First, a pilot experiment was performed using 30 starfish (diameter 6.4–7.5 cm) that had been starved for 1 week to normalise their physiological status. Then animals were injected with different doses of asterotocin (10 μl of 10^−6^–10^−3^ M; *n* = 5 for each dose) or with 10 μl of water (negative control; *n* = 5) or with 10 μl of 10^−3^ M S2 (positive control; *n* = 5). The animals were injected at a site located in the aboral body wall of an arm proximal to the junction with the central disc and adjacent to the madreporite. Care was taken to ensure that the tip of a Hamilton syringe used for injections was pushed through the body wall into the perivisceral coelom but not too deep so as to avoid injecting into the digestive organs. The doses of asterotocin injected were informed by our analysis of the volume of the perivisceral coelomic fluid in *A. rubens*. Thus, injection of 10 μl of 10^−6^–10^−3^ M asterotocin was estimated to achieve concentrations in the perivisceral coelom of ~ 10^−9^, 10^−8^, 10^−7^ and 10^−6^ M, respectively, which are the concentrations at which asterotocin was found to be effective as a cardiac stomach relaxant when tested in vitro. Following injection, the starfish were placed in a glass vessel containing seawater so that the mouth of the animal could be observed from below.

Having established that asterotocin induced cardiac stomach eversion in all of the animals injected with 10 μl 10^−4^ M or 10 μl 10^−3^ M asterotocin, an experiment was performed to examine the time course of asterotocin-induced cardiac stomach eversion in *A. rubens*. For this experiment, 20 adult specimens of *A. rubens* (13.1–17.1 cm in diameter) were selected and starved for 1 week before the experiment. Then, the animals were injected with 10 μl of distilled water or 10 μl of 10^−3^ M synthetic asterotocin. To enable observation of cardiac stomach eversion, following injection, starfishes were placed individually in a petri dish containing 90 ml of artificial seawater. A petri dish was used as it has a shallow depth, which prevented the starfish from climbing vertically and therefore enabling recording of the whole oral surface of the starfish over time using a video camera (Canon EOS 700D) positioned beneath the petri dish. The oral surface of starfish was video recorded for 15 min, and static images from the video recordings were captured at 30-s intervals from the time of injection to 10 min post-injection, during which time maximal stomach eversion was observed. The two-dimensional area of the everted cardiac stomach was measured from the images using the ImageJ software and normalised as a percentage of the area of the central disc of the animal, which was calculated as the area of a circle linking the junctions between the five arms.

While examining the effect of asterotocin in inducing cardiac stomach eversion in *A. rubens*, it was also observed that asterotocin induced arm flexion and/or a “humped” posture resembling the posture that starfish have when feeding on prey. To investigate the influence of this effect of asterotocin on whole-animal behaviour in starfish, experiments were performed to compare the righting behaviour of asterotocin-injected animals with non-injected, water-injected and S2-injected animals. Starfish righting behaviour occurs if they are upturned so that their underside (oral surface) is uppermost. One or more of the arms or rays then twist (active rays) until the tube feet can make contact with and adhere to the floor surface. Then, the other inactive rays and the central disc are flipped over in a somersault-like manoeuvre to bring the entire oral surface back into contact with the floor surface [37, 74]. For an experiment investigating if asterotocin affects the righting behaviour of *A. rubens*, 20 adult animals (10.2–14.9 cm in diameter) were first starved for 1 week prior to the experiment to normalise their physiological status. First, the righting behaviour of starfish without injection was observed, and if righting behaviour did not occur or took more than 5 min, the animal was categorised as unhealthy and unsuitable for the experiment. Following a 30-min recovery period, starfish were either injected with 10 μl of 10^−3^ M asterotocin (*n* = 10) or 10 μl of distilled water (*n* = 10). Then, after 5 min, each starfish was placed with their oral side lowermost in a large glass container fully immersed in artificial seawater for 10 min, after which they were turned upside down with their oral side uppermost and the time taken to right was measured. For consistency, the time taken for righting was determined by noting when the central disc and all five arms touched either the bottom or the side of the glass tank.

To examine the specificity of the effects of asterotocin on starfish righting behaviour, an experiment was performed where the righting time of starfish (5.5–9.6 cm in diameter; starved for 4 weeks prior to testing) was measured before injection (*n* = 60; pooled from the three treatment groups) and after injection with 5 μl 10^−3^ M asterotocin (*n* = 20) or 5 μl 10^−3^ M S2 (*n* = 20) or 5 μl distilled water (*n* = 20). The time taken to right in seconds as well as the percentage difference between righting with and without injection was calculated. The percentage time difference between righting with and without injection in water-injected, asterotocin-injected and S2-injected animals was determined and plotted as a separated box and whiskers graph in Prism 6 (GraphPad 6). The effect of neuropeptides on righting behaviour was analysed statistically using the Mann-Whitney *U* test or a one-way ANOVA test with a post hoc Dunnett’s multiple comparisons test.

## Additional files

Additional file 1: Determination of the structure of asterotocin in *A. rubens* using mass spectrometry (A). LC-ESI-MS/MS analysis of a synthetic peptide (CLVQDCPEG-NH_2_) with the predicted structure of asterotocin reveals that it elutes with a retention time of 29.8 min and the deconvoluted, monoisotopic, singly charged spectrum derived from MS/MS data for this peptide reveals a singly charged species at a m/z of 960.39, consistent with the expected molecular mass. (B) LC-ESI-MS/MS analysis of an extract of *A. rubens* radial nerve cords reveals the presence of a peptide with identical retention time and a spectrum that is very similar to synthetic asterotocin. Accurate mass measurement of the singly charged species of the peptide was determined and mass error observed was 0.0002 Da (0.21 ppm). (TIF 9763 kb)

Additional file 2: *Asterias rubens* VP/OT-type receptor. The nucleotide sequence (lowercase, 1510) of a cDNA encoding the receptor protein (uppercase, 428 amino acid residues) is shown. Primers used for cloning are represented in bold and underlined text. The asterisk denotes the position of the stop codon. The predicted seven transmembrane domains are highlighted in grey within the protein sequence. This cDNA sequence is identical to part of a longer assembled transcript sequence (contig 1122053), which has been submitted to the GenBank database under the accession number MK279533. (TIF 13215 kb)

Additional file 3: Species names and accession numbers for peptide (Table S1) and receptor (Table S2) sequences in Figs. 1c and 2 a, respectively. (DOCX 119 kb)

Additional file 4: Characterisation of antisera to asterotocin and the asterotocin receptor using an enzyme-linked immunosorbent assay (ELISA). (A) Incubation of rabbit antiserum (blue) at dilutions between 1:500 and 1:16,000 with 0.1 nmol of asterotocin antigen peptide per well reveals that the antigen is detected at dilutions between 1:500 and 1:4000 by comparison with absorbance measurements for pre-immune rabbit serum (undiluted; red) with 0.1 nmol antigen peptide (Lys-asterotocin) per well. All data points are mean values from three replicates. (B) Incubation of guinea pig antiserum (green) at dilutions between 1:500 and 1:16,000 with 0.1 nmol of asterotocin receptor antigen peptide per well reveals that the antigen is detected across the full range of dilutions tested by comparison with absorbance measurements for pre-immune guinea pig serum (undiluted; purple) with 0.1 nmol antigen peptide per well. All data points are mean values from three replicates. (TIF 5347 kb)

Additional file 5: Immunohistochemical assessment of the specificity of the asterotocin antiserum (A) Immunostaining in a transverse section of a radial nerve cord that was incubated with asterotocin antiserum (1:1000 dilution). Immunoreactive cell bodies (arrowheads) can be seen in the ectoneural epithelial layer and a dense network of stained fibres (asterisk) can be seen in the neuropile of the ectoneural region of the radial nerve cord. Staining can also be seen in the external epithelial layer of an adjacent tube foot (arrow). (B) Immunostaining in a transverse section of a radial nerve cord, adjacent to the section shown in (A), that was incubated with antiserum (1:1000) that had been pre-absorbed with the asterotocin antigen peptide (200 μM). Note that the majority of the immunostaining seen in (A) is absent in (B), but there is some residual staining in the ectoneural epithelial layer of the radial nerve cord (arrowheads) and in the epithelial layer of the adjacent tube foot (arrow). Therefore, antibodies to asterotocin were affinity-purified from the antiserum and used for the immunohistochemical analysis of asterotocin expression shown in Fig. 4. Abbreviations: Ec, ectoneural region; Hy, hyponeural region; TF, tube foot. Scale bars: (A) and (B) = 40 μm. (TIF 5965 kb)

Additional file 6: Raw values where data are based on smaller sample sizes. Table S1: Raw data for Fig. 7c inset, which compares the relaxing effects of asterotocin and S2 on cardiac stomach preparations; *n* = 5. Table S2: Raw data for Fig. 7d inset, which shows the mean percentage reversal of ACh-induced contraction of apical muscle preparations caused by asterotocin; *n* = 4. (XLSX 33 kb)

Additional file 7: Video recording showing asterotocin-induced cardiac stomach eversion in *A. rubens.* The video on the left shows that no cardiac stomach eversion is observed in a control animal that had been injected with 10 μl water. The video on the right shows that cardiac stomach eversion is observed in an animal that had been injected with 10 μl of 10^−3^ M asterotocin. These videos are representative of the experiments shown in Fig. 8, and static images from these videos are shown in Fig. 8c. The original video recordings were 12 min in length, and these were then sped up to 45-s videos and combined into a single file using Final Cut Pro software. (MP4 12041 kb)

## Data Availability

All datasets generated or analysed during this study are included in this published article [and its supplementary information files] or are available from the corresponding author on reasonable request. Accession numbers for NCBI, GenBank and UniProt peptides for the peptide sequences in Fig. 1c and the receptor sequences used in Fig. 2a can be found in Tables S1 and S2, respectively, in Additional file 3.
