# Supplementary figures and images for: Ancient role of vasopressin/oxytocin-type neuropeptides as regulators of feeding revealed in an echinoderm
Source: BMC Biol. 2019 Jul 31;17:60. doi: 10.1186/s12915-019-0680-2 (PMC6668147; doi:10.1186/s12915-019-0680-2)

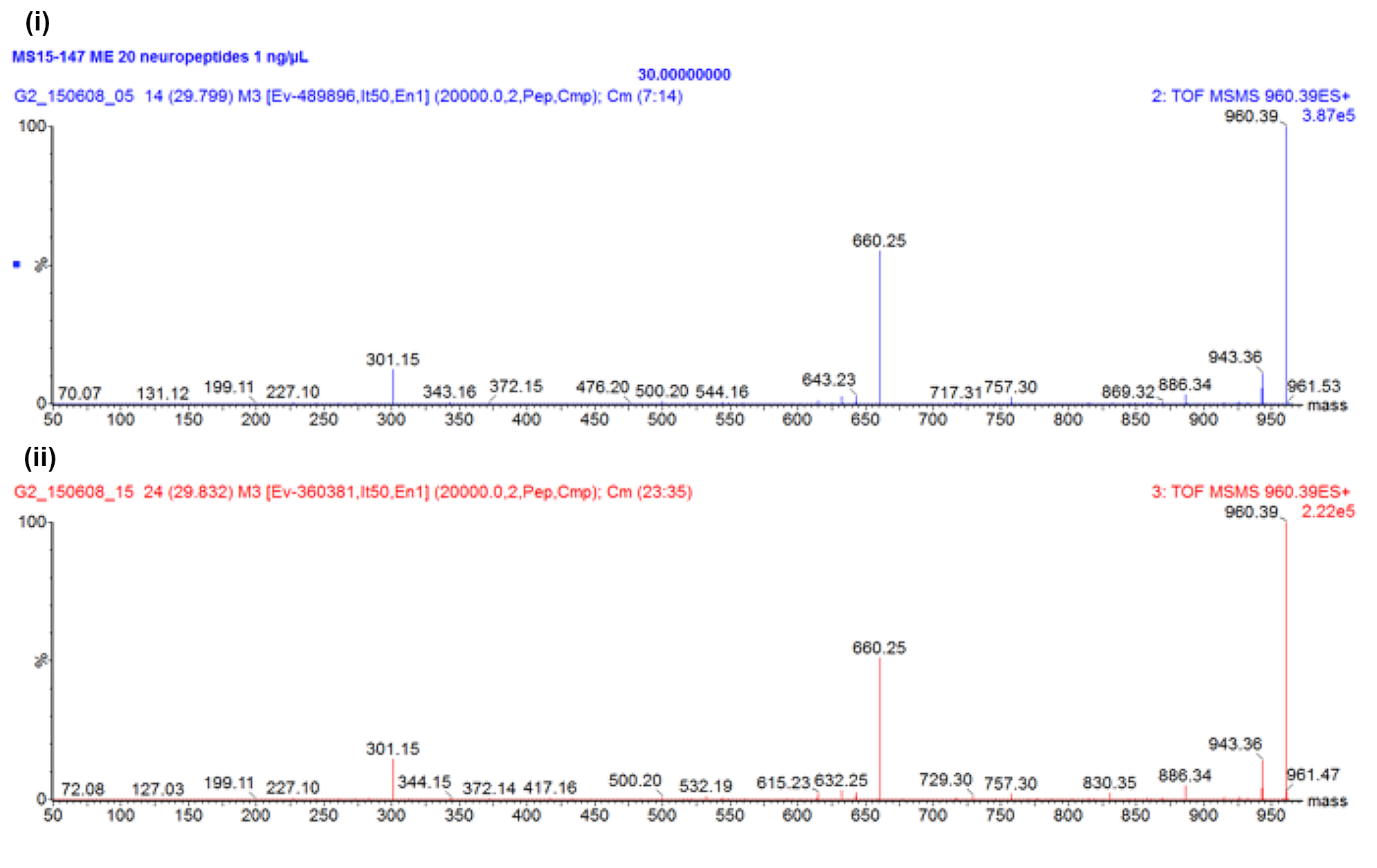

Supplement: Supplementary file 1 — Determination of the structure of asterotocin in A. rubens using mass spectrometry (A). LC-ESI-MS/MS analysis of a synthetic peptide (CLVQDCPEG-NH2) with the predicted structure of asterotocin reveals that it elutes with a retention time of 29.8 min and the deconvoluted, monoisotopic, singly charged spectrum derived from MS/MS data for this peptide reveals a singly charged species at a m/z of 960.39, consistent with the expected molecular mass. (B) LC-ESI-MS/MS analysis of an extract of A. rubens radial nerve cords reveals the presence of a peptide with identical retention time and a spectrum that is very similar to synthetic asterotocin. Accurate mass measurement of the singly charged species of the peptide was determined and mass error observed was 0.0002 Da (0.21 ppm). (TIF 9763 kb) [file 12915_2019_680_MOESM1_ESM.tif]

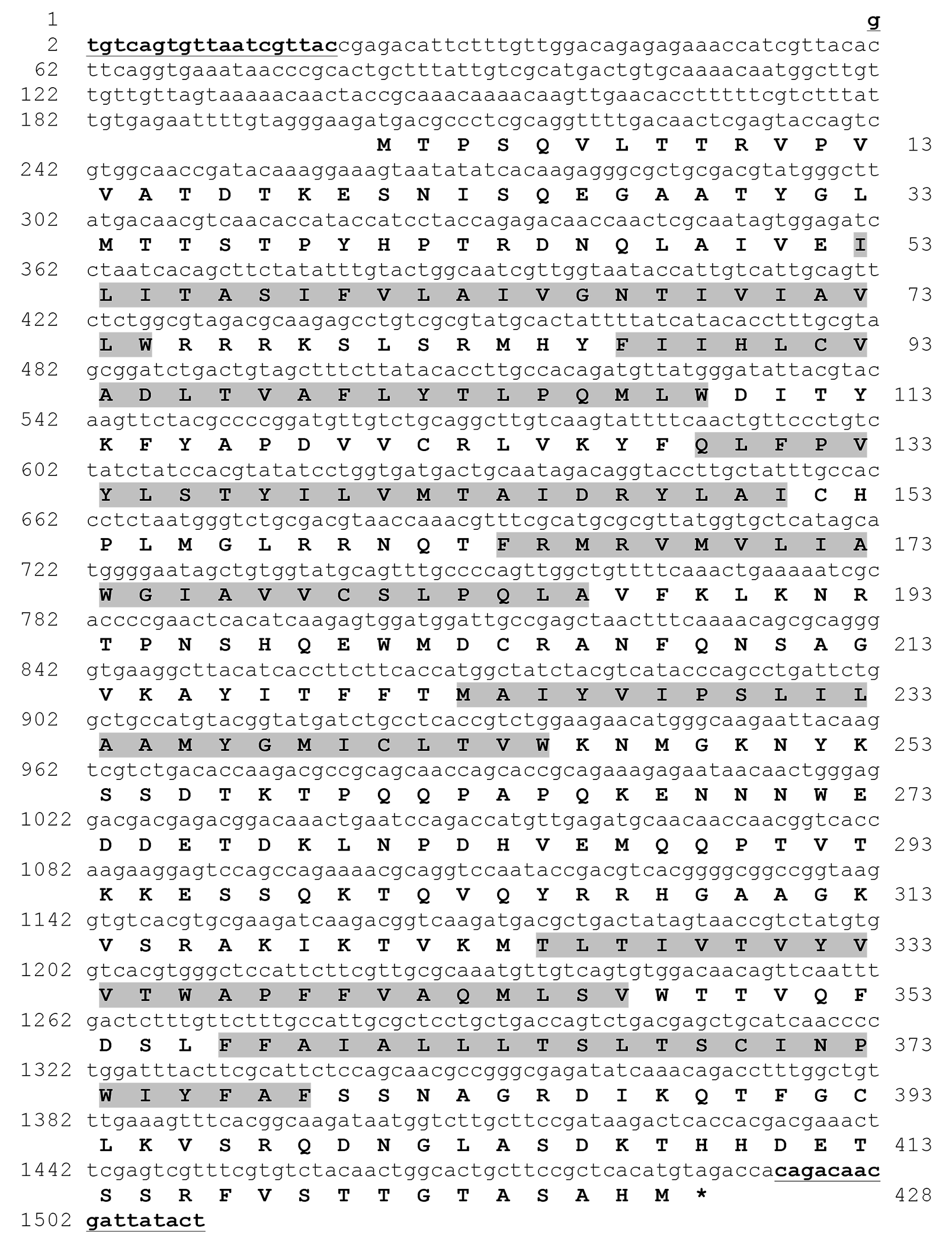

Supplement: Supplementary file 2 — Asterias rubens VP/OT-type receptor. The nucleotide sequence (lowercase, 1510) of a cDNA encoding the receptor protein (uppercase, 428 amino acid residues) is shown. Primers used for cloning are represented in bold and underlined text. The asterisk denotes the position of the stop codon. The predicted seven transmembrane domains are highlighted in grey within the protein sequence. This cDNA sequence is identical to part of a longer assembled transcript sequence (contig 1122053), which has been submitted to the GenBank database under the accession number MK279533. (TIF 13215 kb) [file 12915_2019_680_MOESM2_ESM.tif]

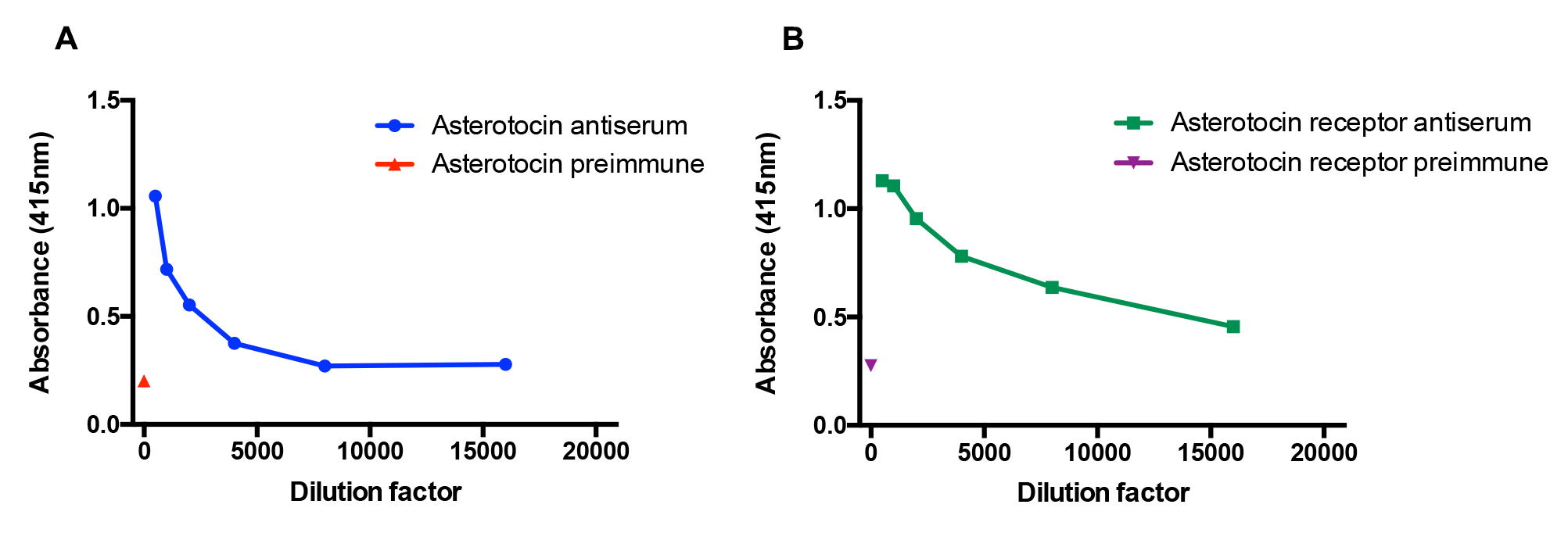

Supplement: Supplementary file 4 — Characterisation of antisera to asterotocin and the asterotocin receptor using an enzyme-linked immunosorbent assay (ELISA). (A) Incubation of rabbit antiserum (blue) at dilutions between 1:500 and 1:16,000 with 0.1 nmol of asterotocin antigen peptide per well reveals that the antigen is detected at dilutions between 1:500 and 1:4000 by comparison with absorbance measurements for pre-immune rabbit serum (undiluted; red) with 0.1 nmol antigen peptide (Lys-asterotocin) per well. All data points are mean values from three replicates. (B) Incubation of guinea pig antiserum (green) at dilutions between 1:500 and 1:16,000 with 0.1 nmol of asterotocin receptor antigen peptide per well reveals that the antigen is detected across the full range of dilutions tested by comparison with absorbance measurements for pre-immune guinea pig serum (undiluted; purple) with 0.1 nmol antigen peptide per well. All data points are mean values from three replicates. (TIF 5347 kb) [file 12915_2019_680_MOESM4_ESM.tif]

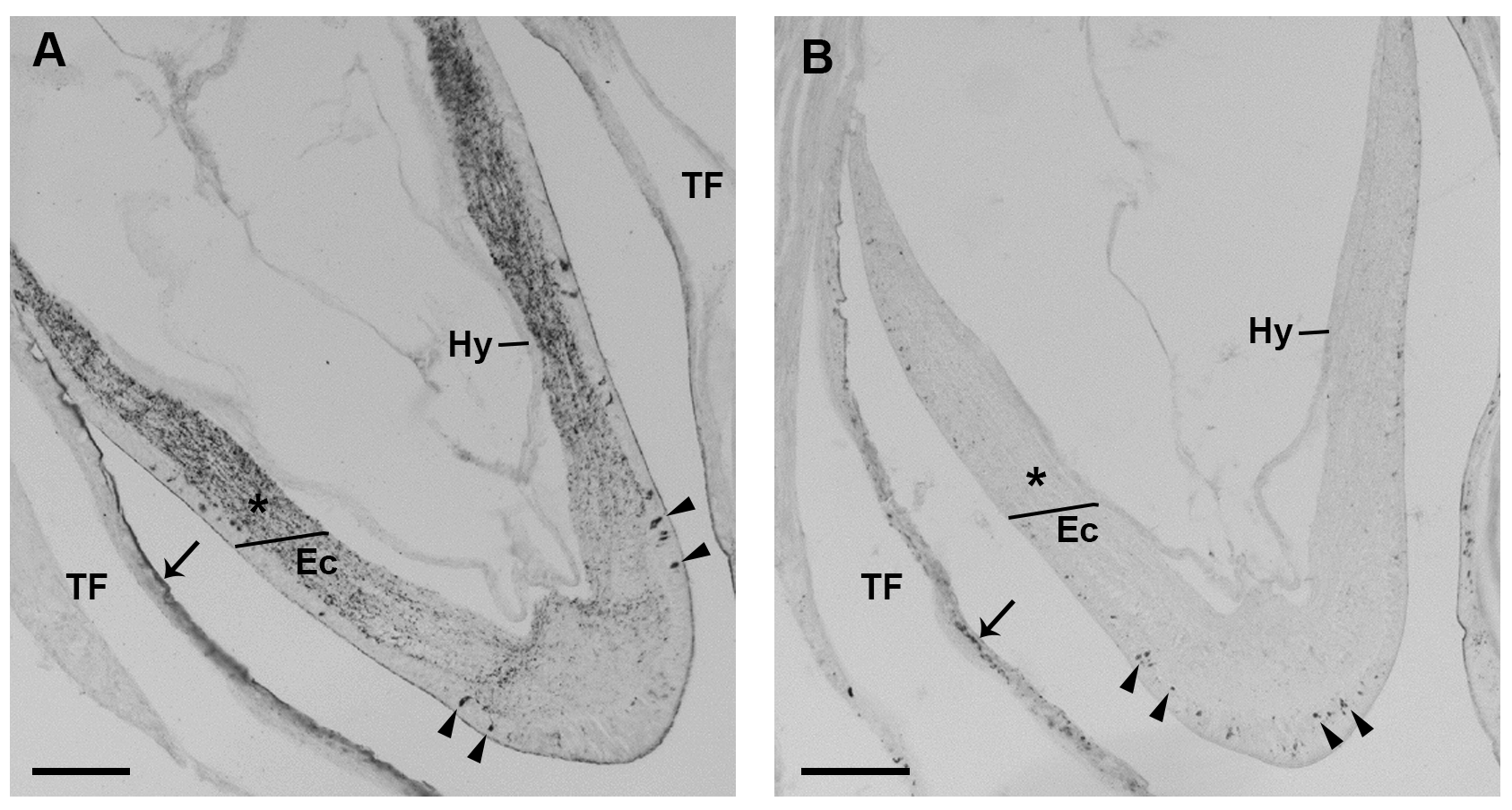

Supplement: Supplementary file 5 — Immunohistochemical assessment of the specificity of the asterotocin antiserum (A) Immunostaining in a transverse section of a radial nerve cord that was incubated with asterotocin antiserum (1:1000 dilution). Immunoreactive cell bodies (arrowheads) can be seen in the ectoneural epithelial layer and a dense network of stained fibres (asterisk) can be seen in the neuropile of the ectoneural region of the radial nerve cord. Staining can also be seen in the external epithelial layer of an adjacent tube foot (arrow). (B) Immunostaining in a transverse section of a radial nerve cord, adjacent to the section shown in (A), that was incubated with antiserum (1:1000) that had been pre-absorbed with the asterotocin antigen peptide (200 μM). Note that the majority of the immunostaining seen in (A) is absent in (B), but there is some residual staining in the ectoneural epithelial layer of the radial nerve cord (arrowheads) and in the epithelial layer of the adjacent tube foot (arrow). Therefore, antibodies to asterotocin were affinity-purified from the antiserum and used for the immunohistochemical analysis of asterotocin expression shown in Fig. 4. Abbreviations: Ec, ectoneural region; Hy, hyponeural region; TF, tube foot. Scale bars: (A) and (B) = 40 μm. (TIF 5965 kb) [file 12915_2019_680_MOESM5_ESM.tif]
