# Supplementary material for: Ancient role of vasopressin/oxytocin-type neuropeptides as regulators of feeding revealed in an echinoderm
Source: BMC Biol. 2019 Jul 31;17:60. doi: 10.1186/s12915-019-0680-2 (PMC6668147; doi:10.1186/s12915-019-0680-2)
Supplement: Supplementary file 3 — Species names and accession numbers for peptide (Table S1) and receptor (Table S2) sequences in Figs. 1c and 2 a, respectively. (DOCX 119 kb) [file 12915_2019_680_MOESM3_ESM.docx]

**Supplementary Table S1.**

| Abbreviations | Full species name | Accession numbers (NCBI) |
| --- | --- | --- |
| *A_rub* | *Asterias rubens* | KT601711 |
| *S_pur* | *Strongylocentrotus purpuratus* | XM_003724442.2 |
| *S_kow* | *Saccoglossus kowalevskii* | FF512954 |
| *B_flo* | *Branchiostoma floridae* | XM_002608950 |
| *C_int* | *Ciona intestinalis* | AB432887 |
| *H_sapVP* | *Homo sapiens* | M25647 |
| *H_sapOT* | *Homo sapiens* | M25650 |
| *E_fet* | *Eisenia fetida* | AB014478 |
| *L_gig* | *Lottia gigantea* | FC764872.1 |
| *D_pul* | *Daphnia pulex* | EFX71881 |
| *T_cas* | *Tribolium casteneum* | NP_001078831 |
| *C_ele* | *Caenorhabditis elegans* | AFJ42491 |

**Supplementary Table S2.**

| Abbreviations | Full species name | Accession numbers |
| --- | --- | --- |
| Hs_V1aR | *Homo sapiens* | NM_000706.4 ^a^ |
| Hs_V1bR | *Homo sapiens* | NM_000707.4 ^a^ |
| Hs_V2R | *Homo sapiens* | NM_000054.5 ^a^ |
| Hs_OTR | *Homo sapiens* | NM_001354655.1 ^a^ |
| Ci_VPOTR | *Ciona intestinalis* | 18586058 ^b^ |
| Sk_VPOTR | *Saccoglossus kowalevskii* | XP_002735546 ^a^ |
| Sp_VPOTR | *Strongylocentrotus purpuratus* | XM_779280.4 ^a^ |
| Ar_VPOTR | *Asterias rubens* | MK279533 ^b^ |
| Ls_VPOTR | *Lymnaea stagnalis* | U27464.1 ^a^ |
| Ef_VPOTR | *Eisenia fetida* | AB121771.1 ^a^ |
| Tc_VPOTR | *Tribolium casteneum* | EU128495.1 ^a^ |
| Hs_NPSR | *Homo sapiens* | AY310326.1 ^a^ |
| Bf_NPSR1 | *Branchiostoma floridae* | XP_002605431 ^a^ |
| Bf_NPSR2 | *Branchiostoma floridae* | XP_002605430 ^a^ |
| Sk_NPSR | *Saccoglossus kowalevskii* | XP_006813812 ^a^ |
| Ar_NGFFYR | *Asterias rubens* | KP171535 ^b^ |
| Sp_NGFFFR | *Strongylocentrotus purpuratus* | KP171538 ^b^ |
| Lg_CCAPR | *Lottia gigantea* | XP_009061331 ^a^ |
| Ct_CCAPR | *Capitella teleta* | ELU12393 ^b^ |
| Dm_CCAPR | *Drosophila melanogaster* | AY219842 ^a^ |
| Tc_CCAPR | *Tribolium casteneum* | NM_001083326 ^a^ |
| Hs_GnRHR | *Homo sapiens* | NP_000397.1 ^b^ |
| Bf_GnRHR1 | *Branchiostoma floridae* | ACC68665.1 ^b^ |
| Bf_GnRHR2 | *Branchiostoma floridae* | ACC68666.1 ^b^ |
| Ar_GnRHR | *Asterias rubens* | KU888680 ^b^ |
| Sp_GnRHR1 | *Strongylocentrotus purpuratus* | NP_001116990.1 ^b^ |
| Sp_GnRHR2 | *Strongylocentrotus purpuratus* | NP_001116992 ^b^ |
| Sp_GnRHR2 | *Strongylocentrotus purpuratus* | NP_001116991 ^b^ |
| Dr_GnRHR1 | *Danio rerio* | NP_001138452 ^b^ |
| Dr_GnRHR2 | *Danio rerio* | NP_001138451 ^b^ |
| Gg_GnRHR1 | *Gallus gallus* | NP_989984 ^b^ |
| Gg_GnRHR2 | *Gallus gallus* | NP_001012627.1 ^b^ |
| Dp_AKHR | *Daphnia pulex* | ACD75498 ^b^ |
| Bm_AKHR | *Bombyx mori* | NP_001037049.1 ^b^ |
| Rp_AKHR | *Rhodnius prolixus* | KF534791 ^b^ |
| Ag_AKHR | *Anopheles gambiae* | ABD60146.1 ^b^ |
| Bm_ACPR | *Bombyx mori* | NP_001127726.1 ^b^ |
| Rp_ACPR | *Rhodnius prolixus* | AKO62856.1 ^b^ |
| Ag_ACPR | *Anopheles gambiae* | ABX52399.1 ^b^ |
| Bf_CRZR3 | *Branchiostoma floridae* | ACC68668.1 ^b^ |
| Bf_CRZR4 | *Branchiostoma floridae* | ACN79527.1 ^b^ |
| Ar_CRZR | *Asterias rubens* | KU888681 ^b^ |
| Sp_CRZR4 | *Strongylocentrotus purpuratus* | XP_011680711.1 ^b^ |
| Sk_CRZR | *Saccoglossus kowalevskii* | XP_006819806.1 ^b^ |
| Lg_CRZR | *Lottia gigantea* | ESP05621 ^b^ |
| Ct_CRZR | *Capitella teleta* | ELT93721.1 ^b^ |
| Ov_CRZR | *Octopus vulgaris* | Q2V2K5 ^b^ |
| Dp_CRZR | *Daphnia pulex* | EFX87464.1 ^b^ |
| Bm_CRZR | *Bombyx mori* | NP_001127719.1 ^b^ |
| Ag_CRZR | *Anopheles gambiae* | AAQ67361.1 ^b^ |

NCBI: a

GenBank: b

Uniprot: c
